# Nanobodies and chemical cross-links advance the structural and functional analysis of PI3Kα

**DOI:** 10.1073/pnas.2210769119

**Published:** 2022-09-12

**Authors:** Jonathan R. Hart, Xiao Liu, Chen Pan, Anyi Liang, Lynn Ueno, Yingna Xu, Alexandra Quezada, Xinyu Zou, Su Yang, Qingtong Zhou, Steve Schoonooghe, Gholamreza Hassanzadeh-Ghassabeh, Tian Xia, Wenqing Shui, Dehua Yang, Peter K. Vogt, Ming-Wei Wang

**Affiliations:** ^a^Department of Molecular Medicine, Scripps Research Institute, La Jolla, CA 92037;; ^b^The CAS Key Laboratory of Receptor Research, Shanghai Institute of Materia Medica, Chinese Academy of Sciences (CAS), Shanghai 201203, China;; ^c^National Center for Drug Screening, Shanghai Institute of Materia Medica, Chinese Academy of Sciences, Shanghai 201203, China;; ^d^iHuman Institute, School of Life Science and Technology, ShanghaiTech University, Shanghai 201210, China;; ^e^School of Artificial Intelligence and Automation, Huazhong University of Science and Technology, Wuhan 430074, China;; ^f^Department of Pharmacology, School of Basic Medical Sciences, Fudan University, Shanghai 200032, China;; ^g^Vlaams Instituut voor Biotechnologie, Nanobody Core, Vrije Universiteit Brussel, 1050 Brussels, Belgium;; ^h^Research Center for Deepsea Bioresources, Sanya 572025, China;; ^i^Department of Chemistry, School of Science, The University of Tokyo, Tokyo 113-0033, Japan

**Keywords:** phosphoinositide 3-kinase (PI3K), nanobody, conformational changes, chemical cross-linking, mass spectrometry

## Abstract

Nanobodies and chemical cross-linking were used to gain information on the identity and positions of flexible domains of PI3Kα. The application of chemical cross-linking mass spectrometry (CXMS) facilitated the identification of the p85 domains BH, cSH2, and SH3 as well as their docking positions on the PI3Kα catalytic core. Binding of individual nanobodies to PI3Kα induced activation or inhibition of enzyme activity and caused conformational changes that could be correlated with enzyme function. Binding of nanobody Nb3-126 to the BH domain of p85α substantially improved resolution for parts of the PI3Kα complex, and binding of nanobody Nb3-159 induced a conformation of PI3Kα that is distinct from known PI3Kα structures. The analysis of CXMS data also provided mechanistic insights into the molecular underpinning of the flexibility of PI3Kα.

## Significance

PI3Kα is a dimeric lipid kinase consisting of a catalytic subunit p110α and a regulatory subunit p85α. It controls cell proliferation and survival and is an important therapeutic target for cancer. However, the development of effective drugs against PI3Kα requires a level of structural information that is currently unavailable, because the extreme flexibility of PI3Kα interferes with structural analysis. Nanobodies were used in conjunction with chemical cross-linking to generate insights into the identity and the positions of the most flexible domains of PI3Kα and into mechanistic aspects of positional flexibility. The studies also reveal the existence of a previously unreported structural conformation of PI3Kα.

Class I phosphoinositol 3-kinases (PI3Ks) are a family of lipid kinases, each composed of a regulatory and a catalytic subunit. Class IA of PI3K consists of three isoforms of the catalytic subunit, p110α, p110β, and p110δ, that are bound to the regulatory subunit p85α or one of its isoforms, p55α, p50α, or p85β (1, 2). The dimer of p110α–p85α is the topic of this investigation and will be referred to as PI3Kα. Class IB contains a single isoform of the catalytic subunit, p110γ, which is associated with the regulatory subunit p101 or p84.

PI3Ks control signaling events that are essential for cell growth and survival, and PI3K activity can be an important factor in tumor formation (2). Starting with the discovery of cancer-specific gain-of-function mutations, PI3Ks became prominent drug targets (3–5). Numerous PI3K inhibitors have been disclosed, but only five are currently approved for therapeutic use (6–10). The field of PI3K inhibitors has moved from so-called “panspecific” compounds toward greater target specificity. The Food and Drug Administration–approved PI3K inhibitors are isoform-specific or isoform-selective. However, since these inhibitors target the wild-type enzymes, toxicities are unavoidable and remain a major clinical problem (11–13). Another strategy is to develop inhibitors that are specific for the cancer-associated mutants of PI3K, and there are encouraging signs that this degree of specificity can be reached (14, 15). Novel chemical strategies that do not rely exclusively on ATP competition also appear promising (16–19). Mutant-specific immunotherapy is now also on the horizon (20).

Detailed structural information could make an important contribution to these efforts. Crystallography, NMR, and hydrogen–deuterium exchange have provided valuable data on large parts but not all of the PI3Kα complex (6, 21–31). The efforts to achieve complete structural data of PI3Ks have recently been complemented by single-particle analysis in cryo–electron microscopy (cryo-EM) (32–34). For PI3Kα, conformational changes associated with inhibition and with activation have been defined (32). However, the positional flexibility of the regulatory subunit domains BH, SH3, and cSH2 prevented the identification of these domains and their positions in the cryo-EM analysis. We have therefore endeavored to use nanobodies and chemical cross-linking to achieve additional insights into the PI3Kα structure, notably the flexible domains. Nanobody binding and chemical cross-linking mass spectrometry (CXMS) provided the identity and docking data for the BH, SH3, and cSH2 domains of the regulatory subunit p85 and revealed information on mechanistic aspects of PI3K flexibility.

## Results

### Generation and Characterization of Nanobodies Binding to Full-Length PI3Kα.

**Fig. 1.**
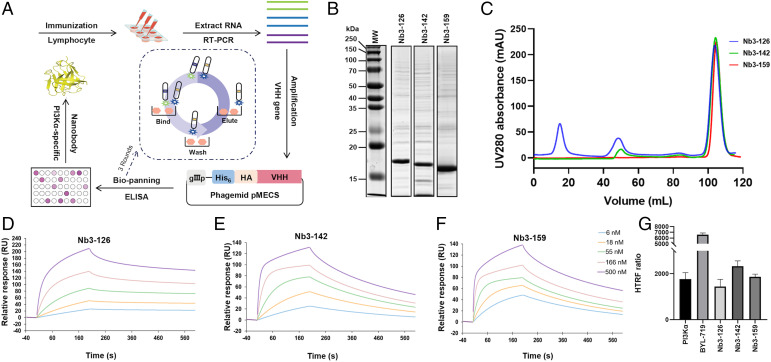
Preparation and characterization of PI3Kα-specific nanobodies. (*A*) Overview of the nanobody generation process. (*B* and *C*) SDS-PAGE/Coomassie blue stain and size exclusion chromatography of the purified nanobodies Nb3-126, Nb3-142, and Nb3-159. (*D*–*F*) SPR sensorgrams demonstrate that nanobodies Nb3-126, Nb3-142, and Nb3-159 bind to PI3Kα with affinities of 12.90, 24.30, and 7.90 nM, respectively. (*G*) HTRF PI 3-kinase assay of purified PI3Kα with indicated nanobodies and inhibitors. Data are from triplicate experiments and reported as median values of three independent measurements.

A llama was immunized, and purified lymphocytes were used to generate a variable domain of heavy chain (VHH) library in phage. Three consecutive rounds of phage display were performed. Each was followed with an ELISA-based binding screen to obtain PI3Kα-specific nanobodies (Fig. 1*A*). From these, 114 colonies were specific for PI3Kα. Based on sequence, 62 full-length nanobodies belonging to 23 different lineages were identified. The identified nanobodies have variable affinity, potency, stability, and expression yield. After selection for expression and purification, a total of 58 nanobodies were obtained with high purity (Fig. 1 *B* and *C*).

### Effect of the Nanobodies on PI3Kα Activity.

The binding kinetics were measured using surface plasmon resonance (SPR) and binding affinities calculated from the kinetic rates (Fig. 1 *D–F* and Table 1). The effect of the nanobodies on PI3Kα activity was assessed using a homogeneous time-resolved fluorescence (HTRF) assay. Based on results of the kinase and affinity assays, three nanobodies, Nb3-126, Nb3-142, and Nb3-159, were selected for additional investigation because of their differing effects on kinase activity. Specifically, nanobody Nb3-159 was selected for its high-affinity binding, although it did not affect PI3Kα activity. Nanobodies Nb3-126 and Nb3-142, however, showed slight activation and inhibition, respectively (Fig. 1*G*).

**Table 1.** Binding and affinity measurements of nanobodies

Nanobody	K_D_ (nM)	K_on_ (10^5^ M^−1^ s^−1^)	K_off_ (10^−3^ s^−1^)
Nb3-126	12.90 ± 1.65	1.73 ± 1.41	2.28 ± 1.81
Nb3-142	24.30 ± 19.70	1.06 ± 0.74	2.58 ± 1.06
Nb3-159	7.90 ± 7.35	3.24 ± 2.51	2.56 ± 1.32

### Coimmunoprecipitation of Nanobodies with p85α Truncation Constructs.

The domains bound by the nanobodies were identified through coimmunoprecipitation (*SI Appendix*, Fig. S1). Truncations of the regulatory domain p85α were expressed in HEK293T cells and pulled down using nanobodies expressed in *Escherichia coli*, purified, and bound to magnetic resin. These experiments showed that Nb3-126 binds to the BH domain, while Nb3-142 and Nb3-159 bind to the nSH2 domain. This is surprising as the BH domain has not previously been identified as having a role in activation.

### Defining Nanobody–PI3Kα Interactions with CXMS.

To validate the results of the coimmunoprecipitations and to explore nanobody-induced conformational and functional changes in PI3Kα, CXMS experiments were performed to obtain the cross-link maps for PI3Kα in complex with different nanobodies. Two complementary amine–amine cross-linkers (BS^3^ and DSG) were chosen to increase the cross-linking coverage of the entire complex. Purified PI3Kα associated with individual nanobody complexes was subjected to chemical cross-linking with either BS^3^ or DSG. The resulting products were evaluated by sodium dodecyl sulfate–polyacrylamide gel electrophoresis (SDS-PAGE) to ensure almost complete cross-linking of PI3Kα with each nanobody (*SI Appendix*, Fig. S2). The reaction products were then digested by trypsin and analyzed by liquid chromatography-tandem mass spectrometry (LC-MS/MS) to map the cross-links within nanobody-bound PI3Kα complexes. Four experimental replicates were performed for each nanobody-bound complex for both BS^3^ and DSG. The CXMS analysis identified a total of 982, 738, and 602 cross-links within the complexes of PI3Kα with Nb3-126 (35, 36), Nb3-142 (37, 38), or Nb3-159 (39, 40), respectively. Among them, 115, 16, and 56 were cross-links between PI3Kα and Nb3-126, Nb3-142, and Nb3-159, respectively (*SI Appendix*, Tables S1–S3). Comparing the cross-links assigned between PI3Kα and different nanobodies, we found both shared and unique residues in the nanobodies that were cross-linked with PI3Kα. For nanobody Nb3-126, the high-frequency cross-links were with Gln1, Ser52, and Lys53; for nanobody Nb3-159, residues Gln1, Ser63, and Lys65 were cross-linked at high frequency with PI3Kα (*SI Appendix*, Fig. S3*C*). The cross-links of nanobody Nb3-142 are located outside the antigen binding domain and therefore do not provide information on the Nb3-142 binding site. The results with nanobodies Nb3-126 and Nb3-159 are in concordance with the coimmunoprecipitations, suggesting these nanobodies bind to the BH and nSH2 domains of p85, respectively.

### Cryo-EM Structures of Nanobody–PI3Kα Complexes.

**Fig. 2.**
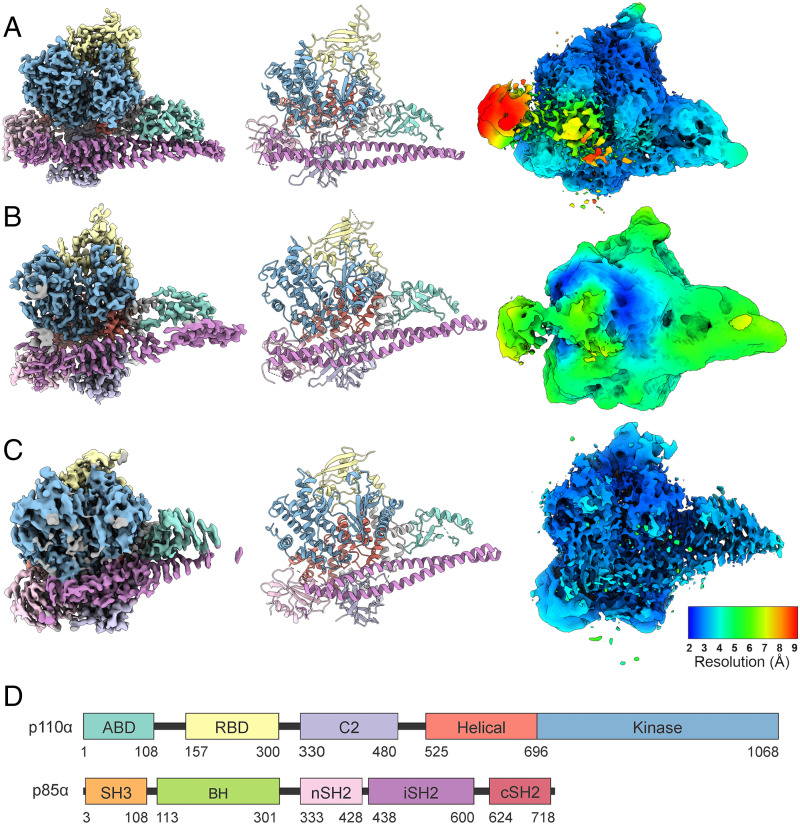
Cryo-EM analysis and modeling of PI3Kα-nanobody complexes. (*A*) Nb3-126, (*B*) Nb3-142, and (*C*) Nb3-159 are shown in three different ways: (*Left*) cryo-EM density, (*Middle*) model, and (*Right*) local resolution plotted on 1% FDR confidence interval. (*D*) Domain structure of p110α and p85α.

**Fig. 3.**
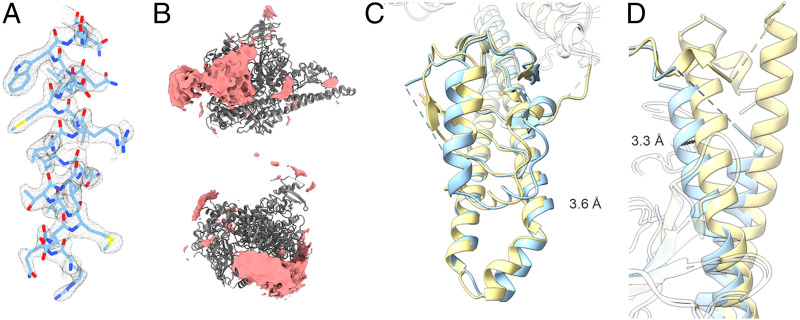
Cryo-EM of PI3Kα-nanobody complexes shows previously unreported features. (*A*) Nb3-126 leads to a high-resolution cryo-EM electron density map as illustrated by a representative α-helix, residues 808 to 826 of p110α. (*B*) Nb3-142 has stabilized extra density as compared with other cryo-EM data. (*C* and *D*) Nb3-159 has an altered position of the ABD and iSH2 domains in comparison with other PI3Kα structures.

The use of nanobodies to stabilize flexible proteins is well established and has been employed previously in the solution of the PI3Kγ structure (33). We have employed nanobodies Nb3-126, Nb3-142, and Nb3-159 in a cryo-EM analysis of PI3Kα (Fig. 2). All three complexes of PI3Kα with nanobodies offer additional insights as compared to the PI3Kα complex alone (*SI Appendix*, Figs. S4–S6 and Table S4). The Nb3-126 complex results in a high-resolution structure of the catalytic core of PI3Kα (Fig. 3*A*), but the extra densities from unmodeled domains of p85α are reduced (Fig. 2*A*). In contrast, the Nb3-142 complex has slightly lower resolution but with much more density from the unmodeled domains (Fig. 3*B*). These changes agree with our previous findings that when inhibited, the unmodeled domains more stably interact with the catalytic core of PI3Kα (32). The Nb3-159 complex, however, differs dramatically from the previous PI3Kα structures with low densities in the ABD and iSH2 regions. The iSH2 is significantly displaced by binding to Nb3-159 as shown in the rmsd map (Fig. 3 *C* and *D*). Yet, the nSH2 domain is stably bound to the helical domain. Since Nb3-159 binding does not result in a detectable change in PI3Kα activity, these structural changes suggest that movement of the ABD and iSH2 domains does not impair catalytic activity.

### Three-Dimensional Variability Analysis of Nanobody–PI3Kα Complexes.

We have performed a three-dimensional (3D) variability analysis (3DVA) of all three nanobody-bound structures using cryoSPARC. In addition to the average structure, cryo-EM can also provide information on the variability of the complex. Previous work has shown that there are a range of displacements of the ABD and iSH2 domains (32). Complexes with nanobodies Nb3-126 and Nb3-142 show analogous variations (*SI Appendix*, Fig. S7).

**Fig. 4.**
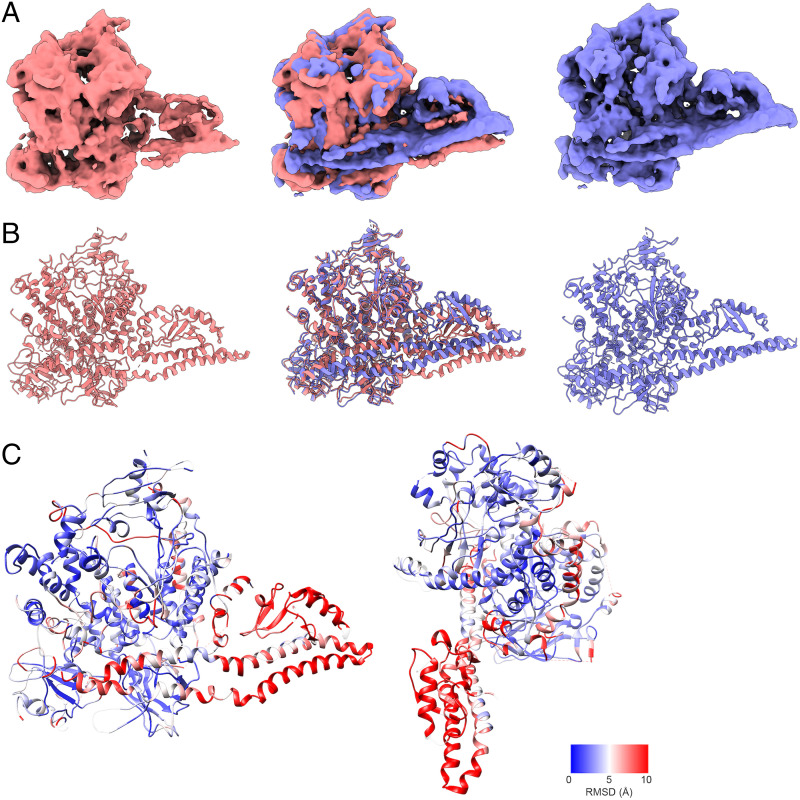
Three-dimensional variability analysis of the complex of PI3Kα with Nb3-159 shows extreme variability of the ABD and iSH2 domains. (*A*) The first component of the Nb3-159 3DVA resolves a motion from a structure similar to the consensus PI3Kα structure (blue) toward a structure with a radically deflected ABD and iSH2 domain (red), and overlay of these structures shows the degree of deflection (*Middle*). (*B*) Models of the two states of component 1 demonstrate a discontinuity in the iSH2 domain that cannot be explained without flexibility or kinking of the coiled-coil domain. (*C*) rmsd values calculated for the Cα positions show that the near edge of the iSH2 is deflected downward, while the far edge is less deflected. There are also changes in the kinase domain N lobes (773 to 777) and C lobes (864 to 874) as well as 803 to 811.

However, with Nb3-159, 3DVA revealed a drastically different situation. Nb3-159 has two components of variability (*SI Appendix*, Fig. S7). At one extreme, Nb3-159 adopts a conformation similar to other PI3Kα complexes. At the opposite extreme (Fig. 4), we find a structure where the iSH2 and ABD are displaced by more than 10 Å and rotated around their long axes, and the nSH2 domain rotates slightly around the p110α-E545–p85α-K379 interaction. The 3D variability electron maps of this unusual conformation have reduced resolution as they are calculated using a fraction of the particles, but despite this limitation, the maps show that iSH2 is no longer a linear coiled-coil domain. Instead, there is a pronounced kink in the iSH2 near the ABD domain. Coiled-coil domains can experience kinking due to mechanical forces (41). We have constructed a model against the 5.0-Å map produced by component 1 of the 3DVA to demonstrate the changes observed. The rmsd data document the extensive changes in the conformation of the PI3Kα complex (Fig. 4*C*). Although the most extreme changes are to the ABD and iSH2, there are significant changes to the kinase domain as well. Loops 773 to 777 and 864 to 874 of the N and C lobes of the kinase domain establish contact, sealing off the active site and inhibiting entry of ATP and substrate. However, this conformation is present only in a minority of particles and thus does not cause a measurable effect on enzyme activity.

It is unclear if this structure can occur without binding of a nanobody, but a similar density map is seen in the 3D classification of Nb3-142 (*SI Appendix*, Fig. S5). This raises the possibility that the ABD and iSH2 domains can adopt conformations that are very different from those seen in crystallography.

### Cryo-EM of Cross-Linked PI3Kα with Nb3-142.

**Fig. 5.**
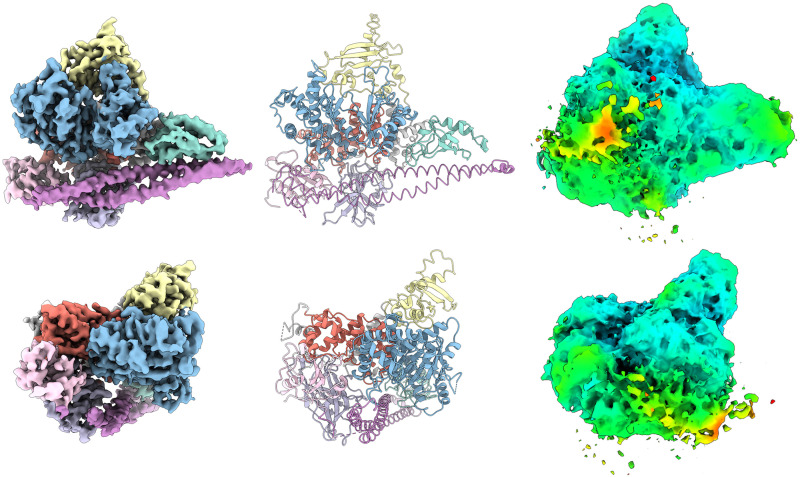
Cryo-EM of DSG cross-linked PI3Kα Nb3-142 complex. Two views of the DSG cross-linked PI3Kα complex with Nb3-142 are shown as (*Left*) electron density, (*Center*) modeling, and (*Right*) 1% FDR confidence surface with local resolution data. Chemical cross-linking allows some previously unresolved regions such as the RBD domain to be stabilized but leads to a decrease in resolution of the unresolved SH3, BH, and cSH2 domain density compared with the un–cross-linked structure. Resolution colors are identical to Fig. 2.

The 3DVA and cryo-EM data both point to flexible conformations of the PI3Kα complex. Using the chemical cross-linking protocol established for the CXMS experiments, we prepared DSG cross-linked PI3Kα with Nb3-142 for cryo-EM analysis (*SI Appendix*, Fig. S8) (42, 43). The resulting structure has a 3.4 Å resolution (Fig. 5). Although chemical cross-linking stabilized some flexible loops that were not present in previous cryo-EM structures of PI3Kα, the SH3, BH, and cSH2 domains as well as the Nb3-142 remained unresolved.

### Docking Unmodeled Domains Using CXMS Data.

**Fig. 6.**
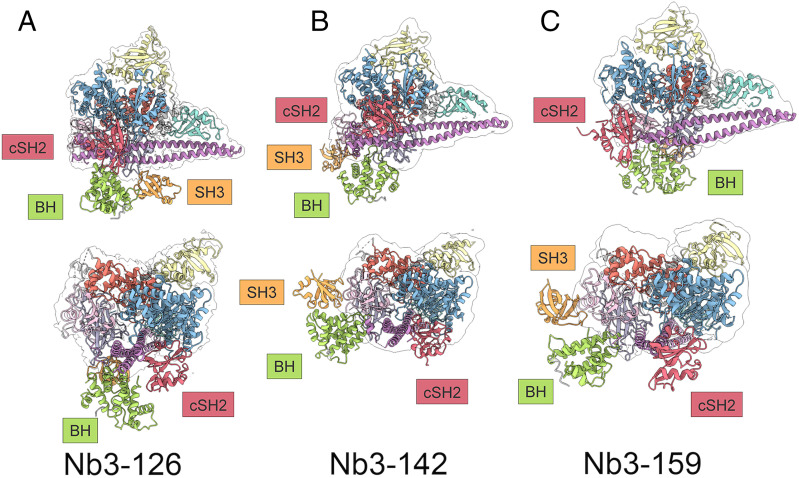
Structural models of PI3Kα bound by (*A*) Nb3-126, (*B*) Nb3-142, and (*C*) Nb3-159. CXMS-derived restraints allow for docking of unresolved domains with the core of PI3Kα in the Nb3-142 stabilized structure. DSG and BS^3^ chemical cross-linking mass spectrometry data were used to construct distance restraints which were used along with existing structural data for the SH3, BH, and cSH2 domains. The cSH2 domain is positioned in front of the kinase domain and blocks access to this site. The BH domain is positioned below the kinase, iSH2, and C2 domains. The position of the SH3 domain is flexible, and docking simulations do not give a consistent position for this domain.

The CXMS results contain cross-links within the PI3Kα complex, which make it possible to extract valuable data on the interactions of PI3Kα domains, notably the unmodeled SH3, BH, and cSH2 domains of p85α. In a previous study, we described three extradensity domains (EDs), ED1, ED2, and ED3, that probably correspond to SH3, BH, and cSH2, respectively. However, these EDs could not be assigned to individual domains of p85α with certainty (32). EDs have been a recurring feature in electron density maps of PI3Kα, and it is therefore important to assign specific domains to these entities. Using the chemical cross-links mapped between the SH3, BH, and cSH2 domains and the modeled domains of PI3Kα, we can now assign specific p85α domains to the EDs. Shown in Fig. 6 *A*–*C* is a model of the complete PI3Kα. Here we apply CXMS-derived restraints and the HADDOCK software program to dock crystal structure models of these p85α domains to the cryo-EM–generated model for Nb3-142–bound PI3Kα. The cSH2 domain binds to the kinase domain and sterically blocks the active site of the enzyme. This finding is in accord with the enzyme-inhibitory activity of Nb3-142. The docked position of the cSH2 domain corresponds to the position of the ED2 seen previously in the BYL-719–bound structure (32). The BH domain is located below the C2, helical, and iSH2 domains and matches the position of the previously described ED3. The cross-links of the SH3 domain are at Y14 and K15 and K80 and K81. They interact with the BH, iSH2, and nSH2 domains of p85α as well as the C2 domain of p110α. These cross-links indicate that the SH3 domain is not exclusively in the position shown in Fig. 6 but can interact with other domains. The SH3 domain corresponds to the previously observed ED1. The structural models of PI3Kα bound by individual nanobodies displayed an overall compatibility of 72 to 81% with the distance restraints of the CXMS analysis. The positional flexibility of PI3Kα domains explains most of the nonsatisfied cross-links. However, there remain some cross-links that are incompatible with our current knowledge of the PI3Kα structure and require additional investigation.

## Discussion

Previous structural studies have analyzed changes of the catalytic subunit p110α induced by the interaction with the regulatory subunit p85α under various experimental conditions. These investigations emphasize a dominant role of the nSH2 domain of p85α in regulating enzyme activity of PI3Kα (44–47). Although work with truncated p85α shows that the BH, SH3, and cSH2 domains are dispensable for enzyme activation via phosphopeptides, in the context of the full-length p85α, the cSH2 domain does exert an enzyme-activating function (44). Specifically, regulation of PI3K by the cSH2 domain only occurs in the presence of the SH3 and BH domains. Cancer-derived mutants of p85α also support the regulatory role of the cSH2 domain (48). The studies presented here complement and extend the understanding of the regulatory functions of these p85 domains.

The persistent technical challenge for the cryo-EM analysis of PI3Kα is the positional flexibility of the BH, SH3, and cSH2 domains of the regulatory subunit p85. The mobility of these domains also creates major obstacles to defining therapeutic targeting sites for allosteric inhibitors. We have used nanobody binding and chemical cross-linking experiments with the goal of stabilizing the domains which are flexible relative to the catalytic core of PI3Kα. While neither of these methods have resulted in a completely stable complex, the acquired CXMS data allowed us to identify interactions between the stable catalytic core and the flexible domains previously named ED1, ED2, and ED3 (32). The analysis of these interactions generated restraints that were used to identify and to dock the domains to the catalytic core and generate a model of PI3Kα. From these data, we conclude that the cSH2 domain is consistently positioned near the opening of the PI3Kα active site and, in this position, would block binding to membranes. It is consistent with the position of the ED2 domain that we previously described (32). This conformation of the cSH2 domain is present in full-length p85α, whereas in truncated versions of p85α it adopts a different conformation (44). Our data further allow the identification and docking positions of the BH (ED3) and SH3 (ED1) domains.

Considering the interactions of the individual nanobodies with PI3Kα, we find stimulating, inhibiting, and neutral effects on enzyme activity. Nanobody Nb3-126 stimulates PI3K activity through interaction with the BH domain. This effect does not involve a displacement of the nSH2 domain. The activation results from a destabilization of the inhibitory cSH2 domain interactions as seen in the electron density confidence map in Fig. 2*A*. The mechanism by which Nb3-126 leads to an increased resolution of the catalytic core of p110α is unclear. Nanobodies and other binding proteins can enhance cryo-EM structure determination through stabilization of flexible regions and by introducing rigid fiducial markers which help particle detection and alignment (49). Nanobody Nb3-126 does neither. Instead, Nb3-126 may cause conformational changes in flexible domains, especially cSH2, which separate them from the catalytic core, thereby reducing interference.

Nanobody Nb3-142 inhibits activity of the complex through binding to the nSH2 domain. This interaction stabilizes the cSH2–catalytic core interactions and could also prevent binding to the cell membrane. The neutral nanobody Nb3-159 binds to the nSH2 domain and induces flexibility in the position and conformation of the ABD and iSH2 domains but causes no net change in activity. The 3DVA shows that binding of Nb3-159 results in a continuum of structures. Most notable is a structure showing extreme conformational changes, especially in the iSH2 and ABD domains but also affecting other domains of p110α. Although this conformation resulted from nanobody binding, there is a possibility that such structures could also be induced by other protein–protein interactions with PI3Kα.

The linkages between domains of p85 are long and a major factor contributing to positional flexibility. The intervening sequences between the SH3, BH, nSH2, iSH2, and cSH2 domains have large, disordered segments and can access a complex 3D space. The domains of p85α are not hinged regions that move in a single plane but rather independent functional domains connected by spaghetti-like peptides. This is also reflected in the CXMS and HADDOCK predictions which show that it is not possible to satisfy all cross-links within a single docked model. Rather, the flexibility generates numerous cross-links, but not all are possible simultaneously. This flexibility may be essential to adopt conformations necessary for binding the diverse upstream receptor tyrosine kinases (RTKs) that initiate the activation of PI3Kα.

We see our work on advancing the structural knowledge of PI3Ks as part of a broader scientific and technical development that is applying cryo-EM to this family of lipid kinases. This includes the analysis of the p110γ–p101 complex of class IB of PI3K (33), the recent investigation on the structure of the class II PI3K isoform PI3KC2α (34), and our initial work on PI3Kα (32). These studies have opened the door to a structural and functional analysis of the full-length PI3K proteins.

## Materials and Methods

### Immunization.

A llama was subcutaneously injected on days 0, 7, 14, 21, 28, and 35 with recombinant human phosphoinositide 3-kinase α (150 µg per injection) and Adjuvant P (GERBU). On day 40, about 100 mL of anticoagulated blood were collected from the llama for lymphocyte preparation.

### Construction of a VHH Library.

A VHH library was constructed from the llama lymphocytes to screen for the presence of antigen-specific nanobodies. To this end, total RNA from peripheral blood lymphocytes was used as a template for first-strand cDNA synthesis with an oligo(dT) primer (ThermoFisher Scientific). The VHH encoding sequences were amplified by PCR using this cDNA, digested with PstI and NotI, and cloned into the PstI and NotI sites of the phagemid vector pMECS (ThermoFisher Scientific). The resulting VHH library is called Core 120 and consists of about 8.5 × 10^8^ independent transformants. Eighty-three percent of the transformants contain the vector with the correct insert size of the VHH-encoding sequences as determined by DNA sequencing.

### Isolation of PI3Kα-Specific Nanobodies.

The Core 120 library was panned for three rounds using solid-phase plates (50) coated with PI3Kα (100 µg/mL in 100 mM NaHCO_3_, pH 8.2). The enrichment of antigen-specific phages was assessed by ELISA screening after each round of panning by comparing the number of phagemid particles eluted from antigen-coated wells with that eluted from negative (uncoated and blocked) control wells (51). This resulted in about 11-fold, 750-fold, and 2,300-fold enrichment after the first, second, and third rounds, respectively. In total, 380 colonies (95 from panning round 1, 95 from round 2, and 190 from round 3) were randomly selected and analyzed by ELISA for the presence of antigen-specific nanobodies in periplasmic extracts.

### Expression and Purification of Nanobodies.

The phagemid vector pMECS with a His_6_ tag and amber stop codon was used for nanobody expression and purification. A 10 mL Luria-Bertani (LB) medium (Beyotime) overnight culture was diluted 1:100 into 1,000 mL fresh TB medium (12 g tryptone [ThermoFisher Scientific], 24 g yeast extract [ThermoFisher Scientific], 4 mL glycerol [SINOPHARM], 2.3 g KH_2_PO_4_ [SINOPHARM], 16.4 g K_2_HPO_4_ [SINOPHARM] per liter) in the presence of 100 µg/mL ampicillin, 2 mM MgCl_2_, and 0.1% glucose at 37 °C. When the culture reached an optical density at 600 nm between 0.6 and 0.9, nanobody expression was induced by 1 mM isopropyl ß-D-1-thiogalactopyranoside (Beyotime), and the temperature was decreased to 28 °C for 16 h. Bacteria were harvested by centrifugation at 5,000 rpm for 25 min, and the pellet was resuspended in 12 mL TES buffer (0.5 M Tris [ABCONE], 0.5 mM EDTA [SINOPHARM], 0.5 M sucrose [Sigma-Aldrich], pH 8.0) by pipetting, followed by shaking on ice for 1.5 h. Eighteen mL TES/4 buffer (TES diluted four times with ddH_2_O) were then added to the suspension and incubated on ice for another 1.5 h with shaking. After centrifuging for 30 min at 35,000 rpm (4 °C), the supernatant was bound to Ni-NTA resin (Cytiva) for 1 h at 4 °C. The resin was subsequently loaded onto a gravity flow column (Sangon Biotech), washed with PBS, and eluted with buffer A (20 mM Tris⋅HCl, pH 8.0, 100 mM NaCl, 300 mM imidazole, 5% [vol/vol] glycerol, and 2 mM β-mercaptoethanol). The eluate was concentrated to 1 mL using an Amicon 10 kDa centrifugal filter (Millipore) and injected into a Superdex 16/60 200 pg gel filtration column (Cytiva) preequilibrated with buffer B (20 mM Hepes, pH 7.6, 100 mM NaCl, and 5 mM DTT). Fractions of gel filtration were collected, concentrated, aliquoted, and kept at −80 °C until use.

### Surface Plasmon Resonance.

The method of SPR was used as previously described (52) using a Biacore 8K instrument with CM5 chips (Cytiva) at room temperature (RT). PI3Kα (1 mg/mL) was diluted to 10 μg/mL with acetate buffer (pH 4.5) and immobilized through a standard amine-coupling protocol with an amine coupling kit (Cytiva). The nanobodies were serially diluted with Biacore EP+ buffer (Cytiva) to five concentrations (500, 166, 55, 18, and 6 nM) to obtain the best-fitting kinetics model. Specifically, the analytes passed through chip surface flow cell 1 (fc1) and flow cell 2 (fc2) at a rate of 30 μL/min. An association phase of 120 s was followed by a dissociation phase of 600 s. The association and dissociation curves of the sensorgrams were analyzed with the Biacore Insight Evaluation (software version 2.0.15.12933), yielding kinetic binding constants. A kinetics rate model using 1:1 binding stoichiometry was employed with nonspecific binding offset using flow cell (fc1) with no PI3Kα conjugation and having a blank control (EP+ buffer).

### Lipid Kinase Activity.

The activity of PI3Kα in the presence of a given nanobody was determined by the PI 3-Kinase HTRF Assay (Millipore) as reported previously (53). Briefly, purified PI3Kα complex was titrated and used at a concentration equivalent to its EC_70_ value (4.8 mM). Nanobody (100 µm, 0.5 μL) was mixed with PI3Kα and PIP_2_ substrate (14.5 μL) and incubated for 10 min before addition of ATP working buffer (5 μL) contained in the kit. The reaction was allowed to proceed for 30 min in the dark at RT before introducing the stop solution and detection mixture. The plates were then incubated for 3 h in the dark and read using an EnVision multilabel plate reader (PerkinElmer).

### Expression and Purification of PI3Kα–Nanobody Complexes.

The methods of PI3Kα expression and purification were described previously (32). PI3Kα–nanobody complexes were formed by incubating PI3Kα and a given nanobody at a molar ratio of 1:1.5 at 4 °C for 30 min. The complex was concentrated to 1 mL using an Amicon 30 kDa centrifugal filter (Millipore) and injected into a Superdex 16/60 200 pg gel filtration column (Cytiva) preequilibrated with buffer B as described above. Fractions of gel filtration were collected, concentrated, aliquoted, and kept at −80 °C until use.

### Cryo-EM Data Acquisition and Image Processing.

The purified PI3Kα–nanobody complex (2.5 μL) at a concentration of 1 mg/mL was applied to glow-discharged holey carbon grids (Quantifoil R1.2/1.3, 300 mesh) and subsequently vitrified by plunging into liquid ethane using a Vitrobot Mark IV (ThermoFisher Scientific). Automatic data collection was performed on a Titan Krios equipped with a Gatan K3 Summit direct electron detector (ThermoFisher Scientific). Dose-fractionated image stacks were subjected to beam-induced motion correction and dose-weighting using MotionCor2.1 (54). A sum of all frames, filtered according to the exposure dose, in each image stack was used for further processing (55). Contrast transfer function parameters for each micrograph were determined by Gctf v1.06 (55). Particle selection and 2D and 3D classifications were performed on a binned dataset using cryoSPARC (v3.0.1) and RELION-3.0-beta2. Particle projections yielded by autopicking through reference-free 2D classifications to discard false-positive particles or particles categorized in poorly defined classes were further processed. They were subjected to consecutive rounds of 3D classification followed by a round of maximum likelihood-based 3D classification, resulting in one or two well-defined subsets with projections. Additional 3D classification with a mask on the complex produced one good subset, which was subsequently subjected to 3D refinement and Bayesian polishing. Local resolution was determined using the Bsoft package (v3.0.1) with half maps as input maps. Three-dimensional variability analysis implemented in cryoSPARC was performed to understand and visualize the dynamics in the PI3Kα–nanobody complexes.

### Chemical Cross-Linking and Protein Digestion.

The Nb3-126 complex was concentrated to 1 mg/mL and cross-linked with 1 mM BS^3^ or DSG (ThermoFisher Scientific) in buffer B for 1 h at 25 °C. For the Nb3-159 and Nb3-142 complexes, 0.5 mg/mL of proteins were prepared and cross-linked with 1 mM BS^3^ or DSG. Cross-linking was quenched by the addition of 20 mM NH_4_HCO_3_ (Sigma) at RT, followed by desalting with PD Spintrap G-25 column (GE Healthcare) and air-dried in a SpeedVac concentrator (Labconco). In each cross-linking condition, the reaction was conducted in four replicates. Cross-linked protein samples were then subjected to SDS-PAGE to evaluate the cross-linking efficiency.

The air-dried samples were resuspended in 8 M urea (Sigma) and 20 mM NH_4_HCO_3_. Subsequently, they were reduced with 5 mM tris(2-carboxyethyl)phosphine (ThermoFisher Scientific) for 20 min at RT and alkylated with 10 mM iodoacetamide (Sigma) for 20 min at RT. The denatured proteins were digested using trypsin (Promega) at an enzyme-to-sample ratio of 1:50 at 37 °C overnight. After being acidified with 1% final concentration of formic acid (Sigma), the supernatant was desalted using UltraMicro Spin Column, Silica C18 (The Nest Group). Finally, peptide samples were lyophilized with a SpeedVac concentrator (Labconco) and redissolved with 0.1% formic acid (FA) before LC-MS/MS analysis.

### LC-MS/MS Analysis and CXMS Data Processing.

LC-MS/MS analysis was carried out on an EASY-nLC 1200 system coupled to a Q Exactive HF mass spectrometer (ThermoFisher Scientific). The peptide samples were loaded onto an analytical column (200 mm × 75 μm) packed with C18-AQ 1.9 μm C18 resin (Dr. Maisch GmbH) at a flow rate of 300 nL/min and separated using a 120 min gradient: 0 to 30 min from 5 to 15% B, 30 to 70 min from 15 to 25% B, 70 to 110 min from 25 to 40% B, 110 to 115 min from 40 to 100% B, and 115 to 120 min 100% B (80% ACN, 0.1% FA as mobile phase B). The top 20 most intense precursor ions from each full scan (resolution 120,000, automatic gain control (AGC) target 3e6, scan range 300 to 1,800 mass-to-charge ratio) were isolated for higher-energy collisional dissociation MS2 analysis (resolution 30,000, AGC target of 1e5) with a dynamic exclusion time of 30 s. Precursors of charge states 3+ to 8+ were selected for MS2 data acquisition.

Software pLink 2.3.9 (56) was used to identify cross-linked peptides, with the data search parameters set as follows: peptide mass 600 to 6,000, peptide length 6 to 60, precursor mass tolerance 10 ppm, fragment mass tolerance 20 ppm, carbamidomethyl [C] and oxidation [M] as variable modifications, and maximum number of missed cleavages as two. The results were filtered by applying a cutoff of False Discovery Rate (FDR) < 5% and E value < 0.001.

### Model Building.

The cryo-EM structure of PI3Kα (Protein Data Bank [PDB] ID 7MYN) (32) was docked into the three cryo-EM maps using phenix.dock_in_map (v1.19-2-4158) (57). Portions of the structure were modeled based upon X-ray crystal structures, including PDB ID 4OVU. Maps were density-modified using phenix.resolve (58). Coordinates were refined in real space using phenix.real_space_refine (59), and maps were sharpened using phenix.autosharpen (60) and Coot (0.9.2-pre EL) (61). Model validation was performed using phenix.validation_cryoem (62) which employs MolProbity (63).

### Figure Preparation.

Figures were produced using Chimera (64) and ChimeraX 1.3 (65). Protein alignments between models were produced using Chimera MatchMaker (66) using the catalytic core of p110α (residues 108 to 1047).

### Generation of Docked Domain Models.

A model of the full-length PI3Kα complex was generated using the Nb3-142–bound structure, the Nb3-142 CXMS data, and the SH3 (PDB ID 1PNJ), BH (PDB ID 6D82), and cSH2 (PDB ID 1QAD) domains. Probable interactions were determined with DisVis 2.0 using the Nb3-142 model with each domain individually, employing the full set of cross-links (67). Proteins were docked using HADDOCK2.4 (68, 69). A total of 1,000 models were generated, and the top three clusters are shown. The cross-links for each complex are summarized in Dataset S1.

### Coimmunoprecipitation.

Nanobody sequences were synthesized using G-block synthesis (IDT). These were cloned into the NcoI and SalI sites of pET-28a (Novogene). The resulting plasmids were transformed into BL21 (DE3). Bacteria were grown to an OD600 of 0.5 in LB medium (BD Biosciences), induced with 1 mM IPTG (Sigma-Aldrich), and incubated for 4 h at 37 °C. The bacteria were harvested by centrifugation at 6,000 × *g* for 10 min, resuspended in 50 mM Tris⋅HCl, pH 7.0, 5% glycerol, and 50 mM NaCl, and lysed using sonication. The resulting suspension was then centrifuged at 15,000 × *g* for 1 h at 4 °C. The supernatant was purified using His-Trap FF column (Cytiva) and eluted with 300 mM imidazole in 50 mM Tris⋅HCl, pH 7.0, 5% glycerol, and 50 mM NaCl. Using Amicon Ultra 10 kDa centrifugal filters (Millipore), the buffer was exchanged to 50 mM Tris⋅HCl, pH 7.0, 5% glycerol, and 50 mM NaCl.

The purified protein was bound to DynaBeads His-Tag (Life Technologies) and rinsed three times with lysis buffer (20 mM Tris, pH 7.5, 100 mM NaCl, 0.5% Nonidet P-40, 0.5 mM PMSF, 0.5% Protease Inhibitor Mixture [Roche]). The PI3Kα complex was produced in HEK293T cells by transfection of pHIV constructs using Lipofectamine 3000 (ThermoFisher Scientific). After 48 h, cells were lysed using lysis buffer. The PI3Kα complex consisting of full-length p110α with N-terminal HA-tagged p85α full-length or residue ranges 75 to 720, 307 to 720, or 1 to 608 was mixed with the prepared nanobody-bound DynaBeads by incubation for 10 min at RT with agitation. These complexes were then washed three times with wash buffer (20 mM Tris, pH 7.5, 150 mM NaCl, 0.1% Nonidet P-40, 0.1 mM PMSF 1× Protease Inhibitor Mixture) and eluted using wash buffer with 300 mM imidazole, separated on an SDS-PAGE gel, and blotted to PVDF (Millipore). The resulting blots were probed with anti-His tag and anti-HA tag antibodies (Cell Signaling) to determine binding.

## Supplementary Material

Supplementary File

Supplementary File

## Data Availability

The electron density maps and atomic coordinates for PI3Kα with nanobodies Nb3-126, Nb3-142, Nb3-159, and Nb3-142 with cross-linking have been deposited in Electron Microscopy Data Bank [https://www.ebi.ac.uk/pdbe/emdb/, accession nos. EMD-27327 (35), EMD-27334 (37), EMD-27330 (39), and EMD-27336 (42), respectively) and PDB (https://www.rcsb.org, accession nos. 8DCP (36), 8DD4 (38), 8DCX (40), and 8DD8 (43), respectively].
