# Supplementary material for: Nanobodies and chemical cross-links advance the structural and functional analysis of PI3Kα
Source: Proc Natl Acad Sci U S A. 2022 Sep 12;119(38):e2210769119. doi: 10.1073/pnas.2210769119 (PMC9499577; doi:10.1073/pnas.2210769119)
Supplement: Supplementary File [file pnas.2210769119.sapp.pdf]

PNAS

Supplementary Information for

Nanobodies and chemical cross-links advance the structural and functional analysis of PI3Kα

Jonathan R. Hart, Xiao Liu, Chen Pan, Anyi Liang, Lynn Ueno, Yingna Xu, Alexandra Quezada, Xinyu Zou, Su Yang, Qingtong Zhou, Steve Schoonooghe, Gholamreza Hassanzadeh-Ghassabeh, Tian Xia, Wenqing Shui, Dehua Yang, Peter K. Vogt, Ming-Wei Wang

Corresponding authors: Wenqing Shui, Dehua Yang, Peter K. Vogt, Ming-Wei Wang
Email: shuiwq@shanghaitech.edu.cn, dhyang@simm.ac.cn, pkvogt@scripps.edu, mwwang@simm.ac.cn

This PDF file includes:

Figures S1 to S8
Tables S1 to S4

Other supplementary materials for this manuscript include the following:

Dataset S1 – Excel file

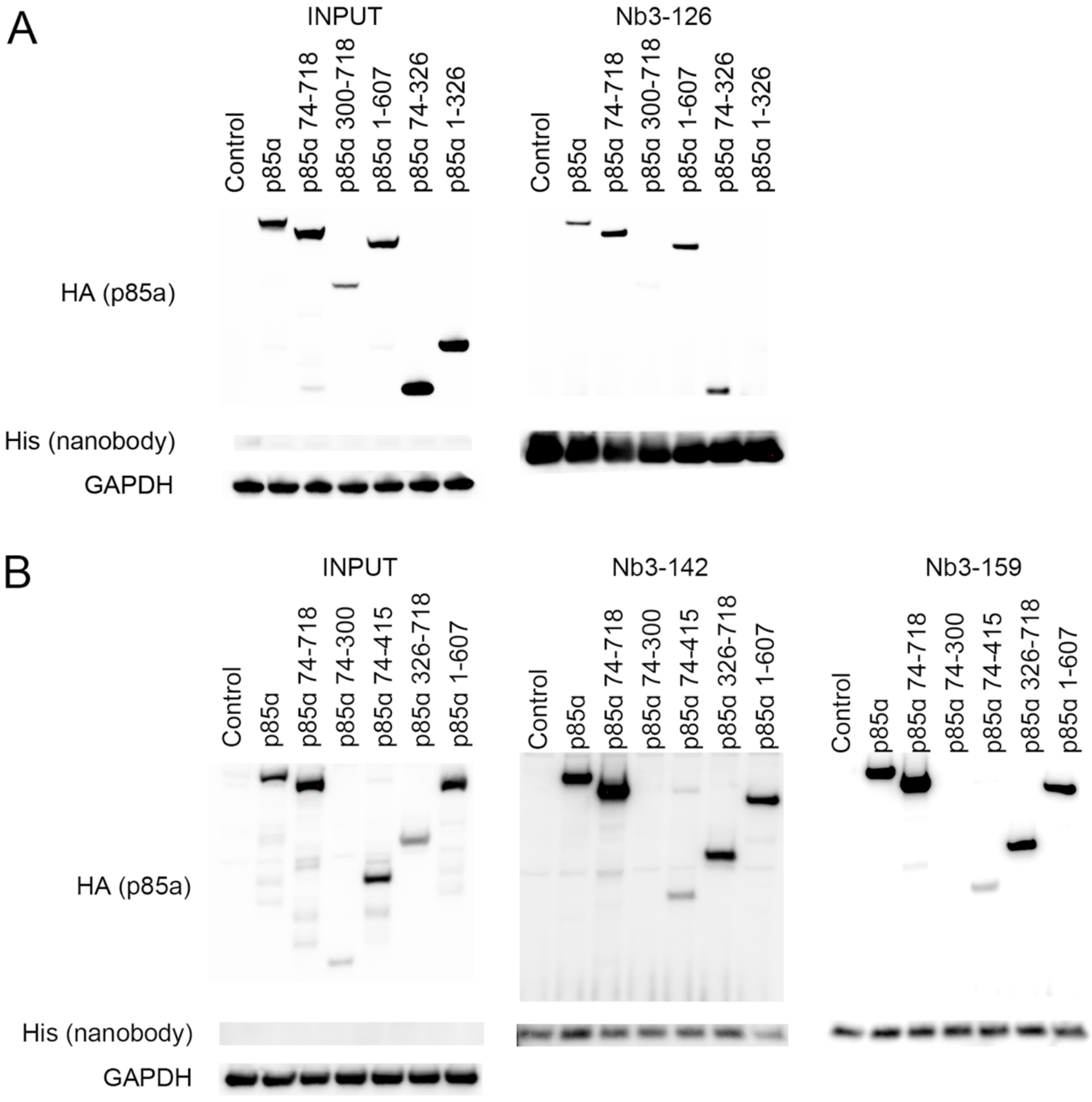

Fig. S1. Co-immunoprecipitation of p110α with indicated N-terminally HA-tagged p85α truncation constructs. (A) Nb3-126 specifically interacts with p85α 74-326, the BH domain. (B) Nb3-142 and Nb3-159 specifically interact with the nSH2 domain in the 326-415 region.

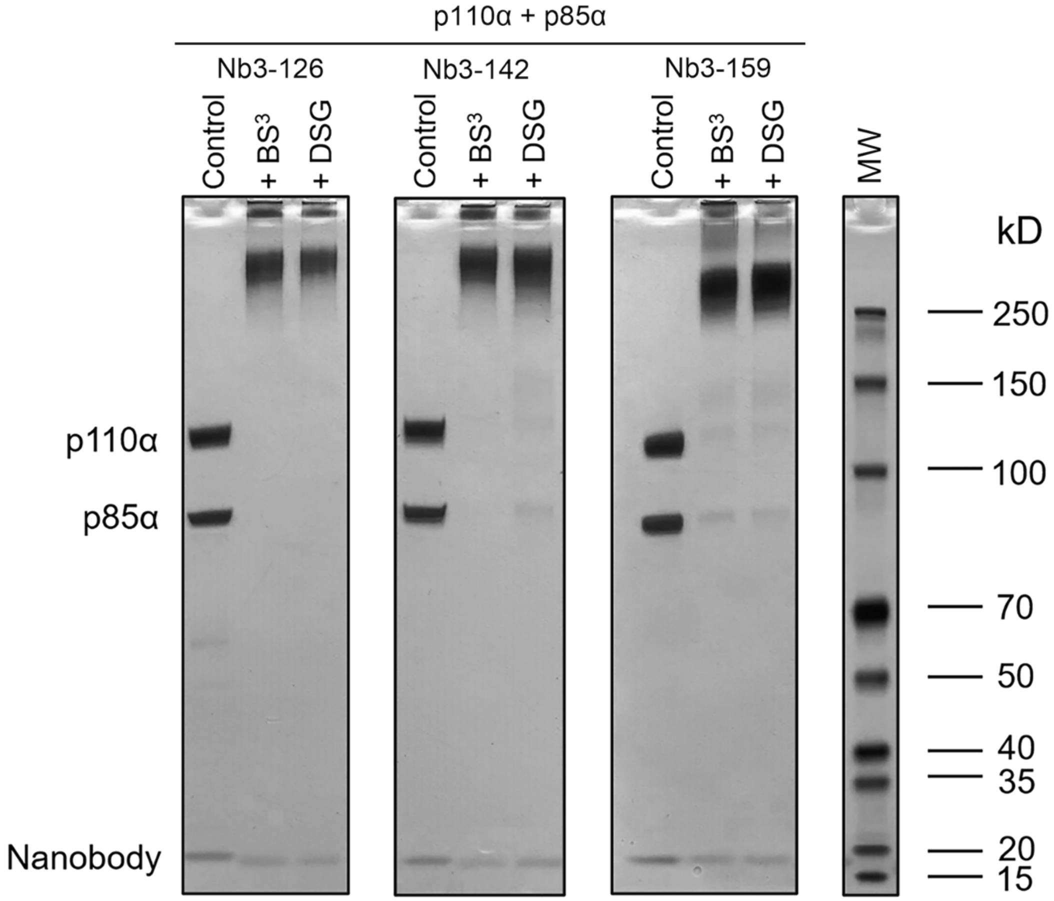

Fig. S2. Cross-linking products of PI3Kα with Nb3-126, Nb3-142 and Nb3-159 were evaluated using SDS-PAGE.

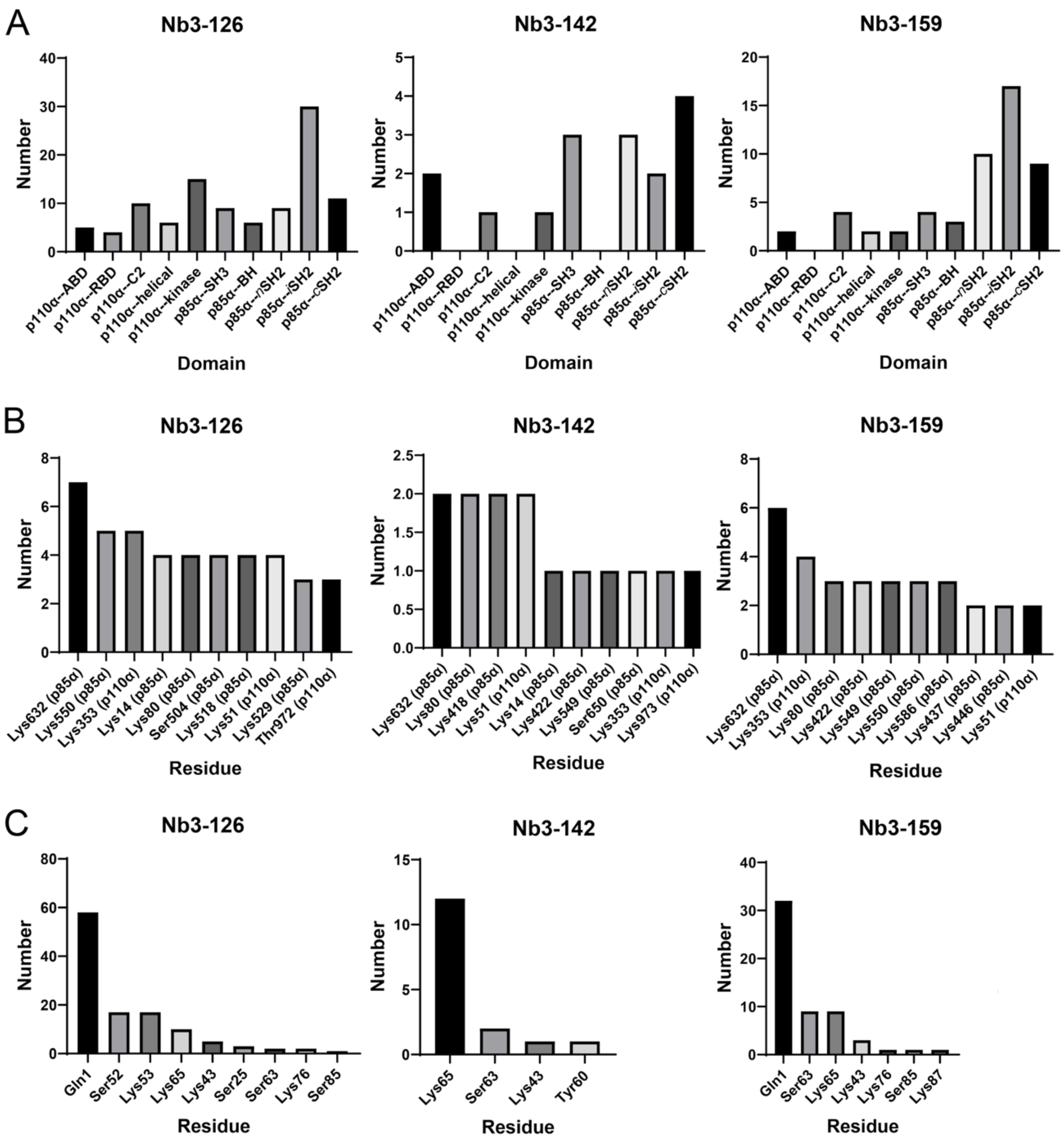

Fig. S3. Cross-links assigned between sites of PI3Kα and the three nanobodies. (A) The number of identified cross-links between each domain of PI3Kα and the nanobodies. (B) The top 10 residues in PI3Kα having the largest number of cross-links with the nanobodies. (C) The number of identified cross-links between a specific residue of the nanobody and that of PI3Kα.

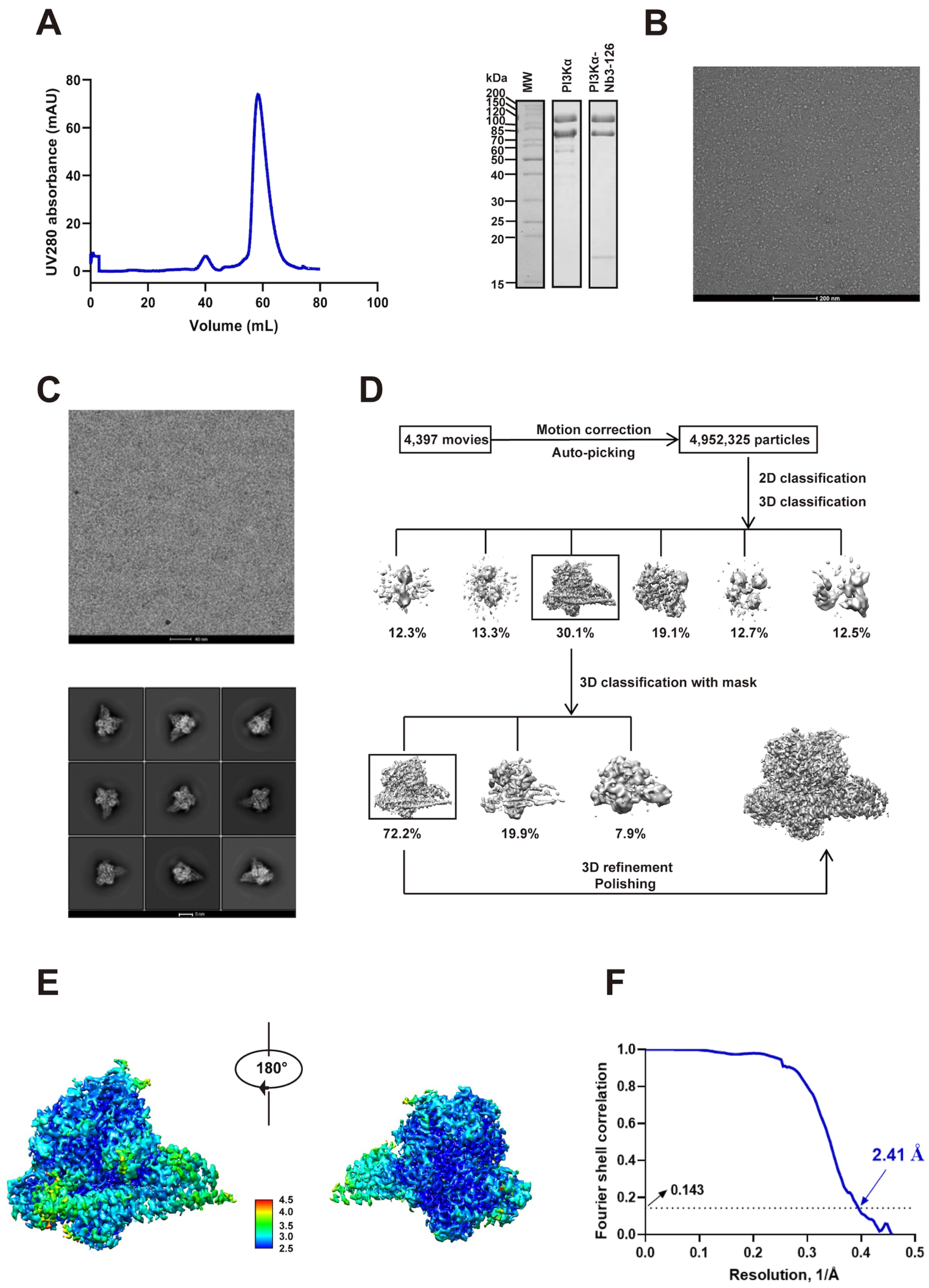
A
B
C
D
E
F
UV280 absorbance (mAU)
Volume (mL)
0
20
40
60
80
100
kDa
MW
PI3Kα
PI3Kα-Nb3-126
200
150
120
100
85
70
60
50
40
30
25
20
15
200 nm
4,397 movies
Motion correction
Auto-picking
4,952,325 particles
2D classification
3D classification
12.3%
13.3%
30.1%
19.1%
12.7%
12.5%
3D classification with mask
72.2%
19.9%
7.9%
3D refinement
Polishing
180°
4.5
4.0
3.5
3.0
2.5
Fourier shell correlation
Resolution, 1/Å
0.143
2.41 Å
1.0
0.8
0.6
0.4
0.2
0.0
0.1
0.2
0.3
0.4
0.5

Fig. S4. Characterization and cryo-EM analysis of the Nb3-126 complex. (A) Analytical size exclusion chromatography, SDS-PAGE/Coomassie blue stain of the purified Nb3-126 complex. (B) Representative negative staining image of the purified Nb3-126 complex. (C) Representative cryo-EM micrographs of the Nb3-126 complex. Representative 2D class averages showing distinct secondary structure features from different views. (D) Workflow of cryo-EM data processing for the Nb3-126 complex. (E) Cryo-EM maps are colored by local resolution (Å). (F) 'Gold-standard' FSC curves of the Nb3-126 complex, indicating the resolution is 2.41 Å at a FSC of 0.143.

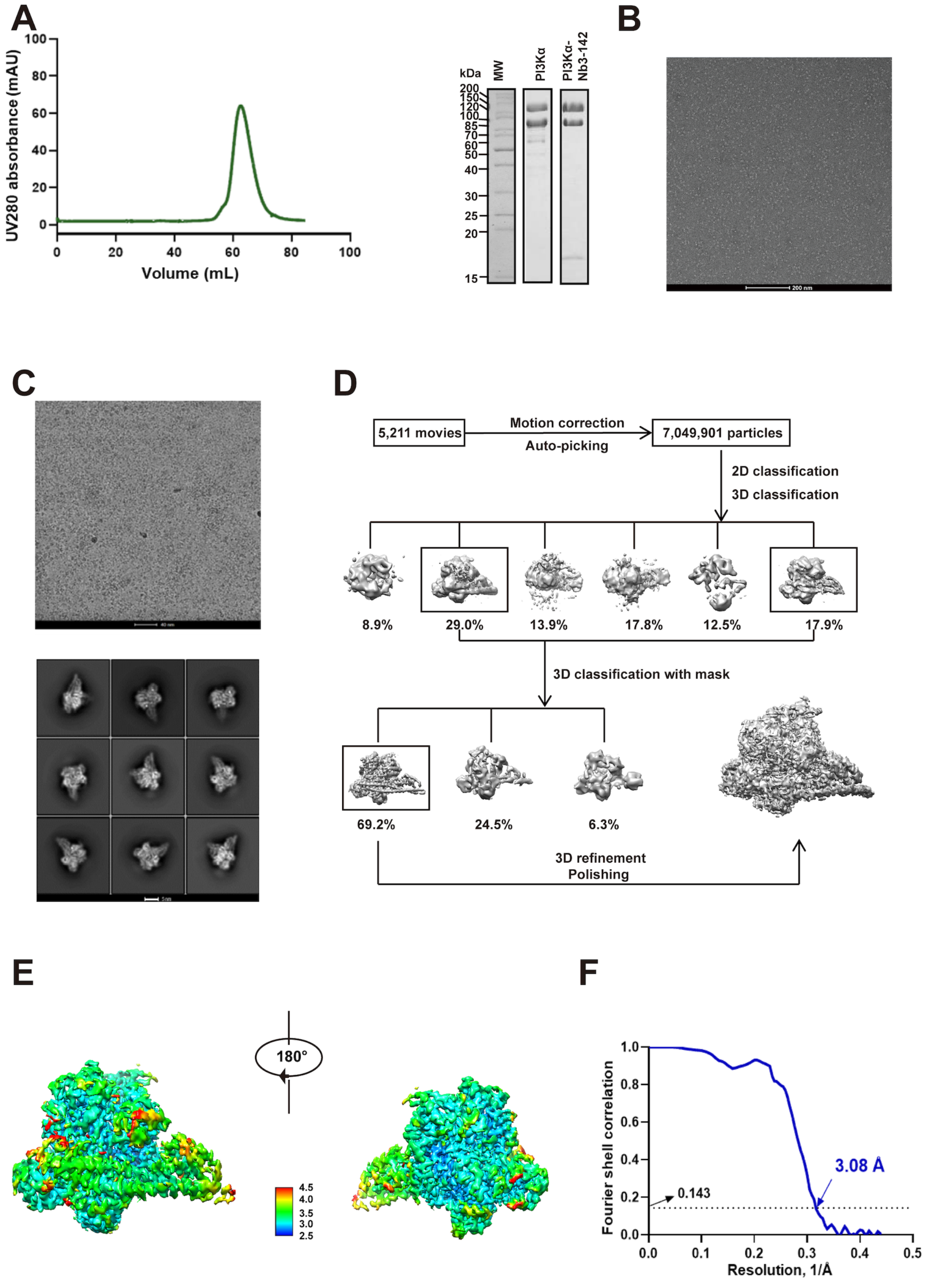

A
UV280 absorbance (mAU)
Volume (mL)
kDa
MW
PI3Kα
PI3Kα-Nb3-142
B
200 nm
C
D
5,211 movies
Motion correction
Auto-picking
7,049,901 particles
2D classification
3D classification
8.9%
29.0%
13.9%
17.8%
12.5%
17.9%
3D classification with mask
69.2%
24.5%
6.3%
3D refinement
Polishing
E
180°
4.5
4.0
3.5
3.0
2.5
F
Fourier shell correlation
3.08 Å
0.143
Resolution, 1/Å

Fig. S5. Characterization and cryo-EM analysis of the Nb3-142 complex. (A) Analytical size exclusion chromatography, SDS-PAGE/Coomassie blue stain of the purified Nb3-142 complex. (B) Representative negative staining image of the purified Nb3-142 complex. (C) Representative cryo-EM micrographs of the Nb3-142 complex. Representative 2D class averages showing distinct secondary structure features from different views. (D) Workflow of cryo-EM data processing for the Nb3-142 complex. (E) Cryo-EM maps are colored by local resolution (Å). (F) 'Gold-standard' FSC curves of the Nb3-142 complex, indicating the resolution is 3.08 Å at a FSC of 0.143.

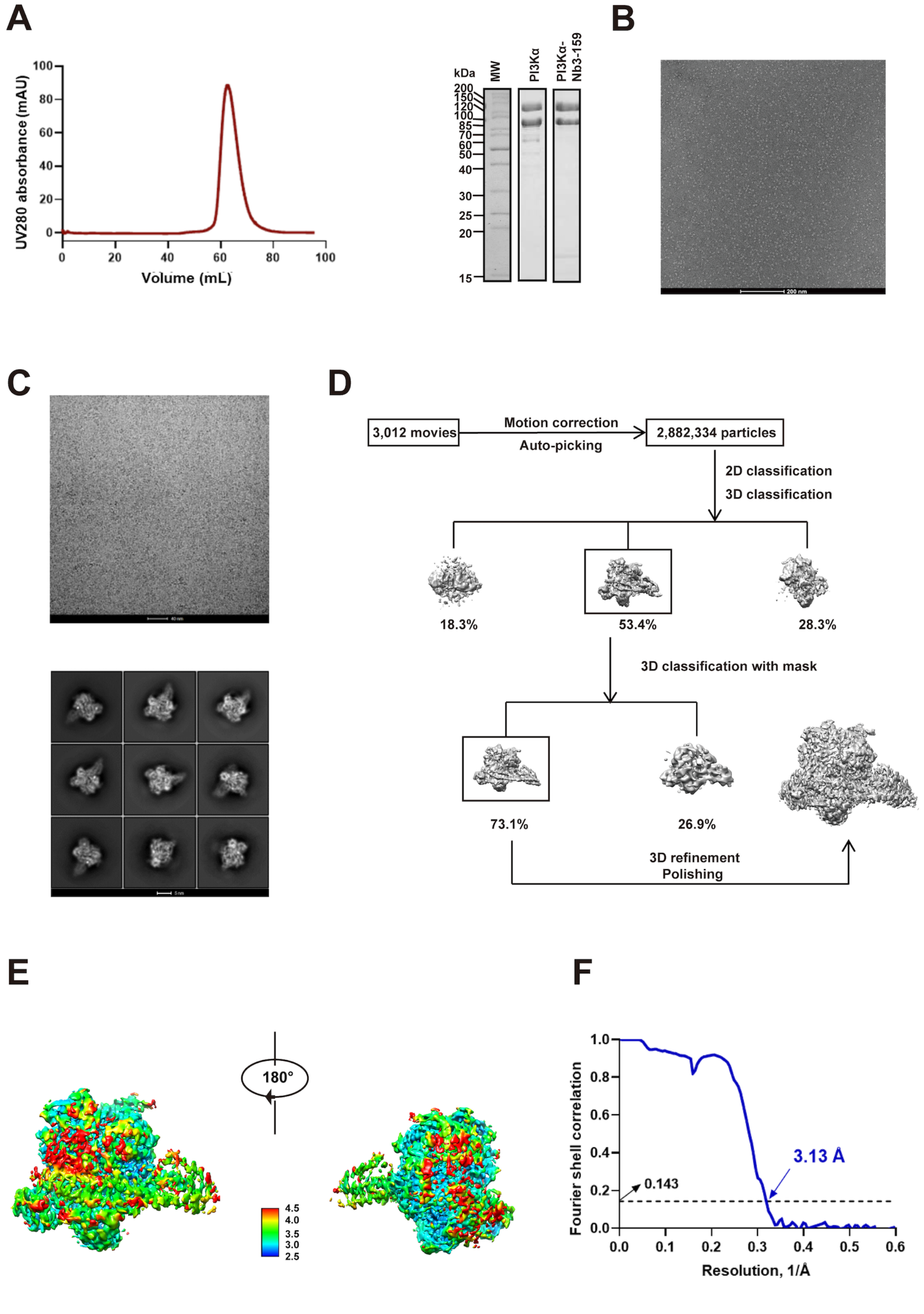

Fig. S6. Characterization and cryo-EM analysis of the Nb3-159 complex. (A) Analytical size exclusion chromatography, SDS-PAGE/Coomassie blue stain of the purified Nb3-159 complex. (B)

Representative negative staining image of the purified Nb3-159 complex. (C) Representative cryo-EM micrographs of the Nb3-159 complex. Representative 2D class averages showing distinct secondary structure features from different views. (D) Workflow of cryo-EM data processing for the Nb3-159 complex. (E) Cryo-EM maps are colored by local resolution (Å). (F) 'Gold-standard' FSC curves of the Nb3-159 complex, indicating the resolution is 3.13 Å at a FSC of 0.143.

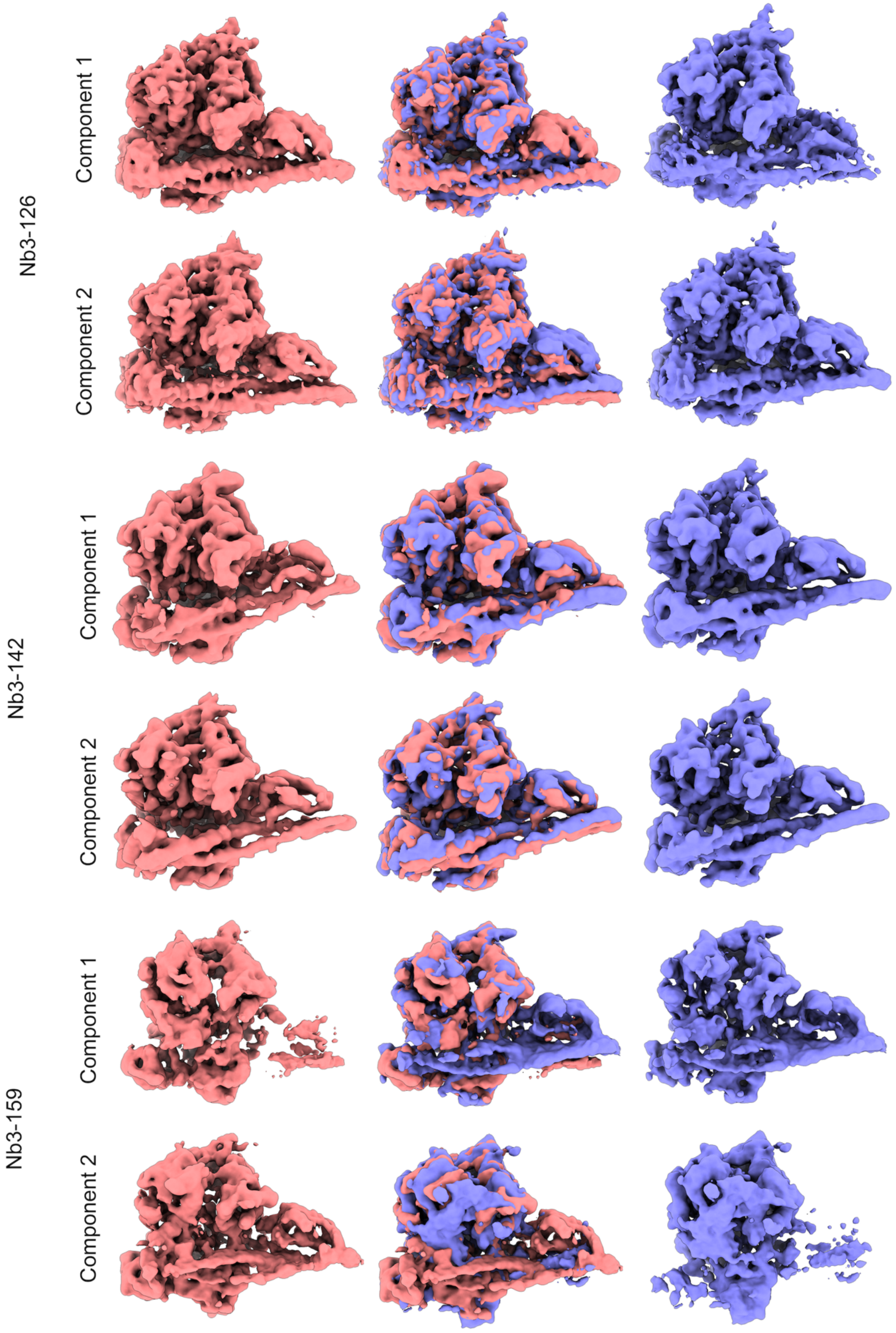
Nb3-126
Component 1
Component 2
Nb3-142
Component 1
Component 2
Nb3-159
Component 1
Component 2

Fig. S7. 3D variability analysis of Nb3-126, Nb3-142, and Nb3-159. The indicated complexes are shown as positive (red) and negative (blue) variations with the components displayed. Nb3-126 and Nb3-142 both show two motions of the ABD and iSH2 corresponding to a side-to-side and up-and-down motion of the ABD-iSH2 region. However, Nb3-159 shows a radical deflection of the ABD-iSH2 domain in component 1 positive (red) and near complete disorder of the ABD and iSH2 domains in the component 2 negative (blue). Both of these motions are accompanied by major structural changes in the kinase domain including narrowing of the active site.

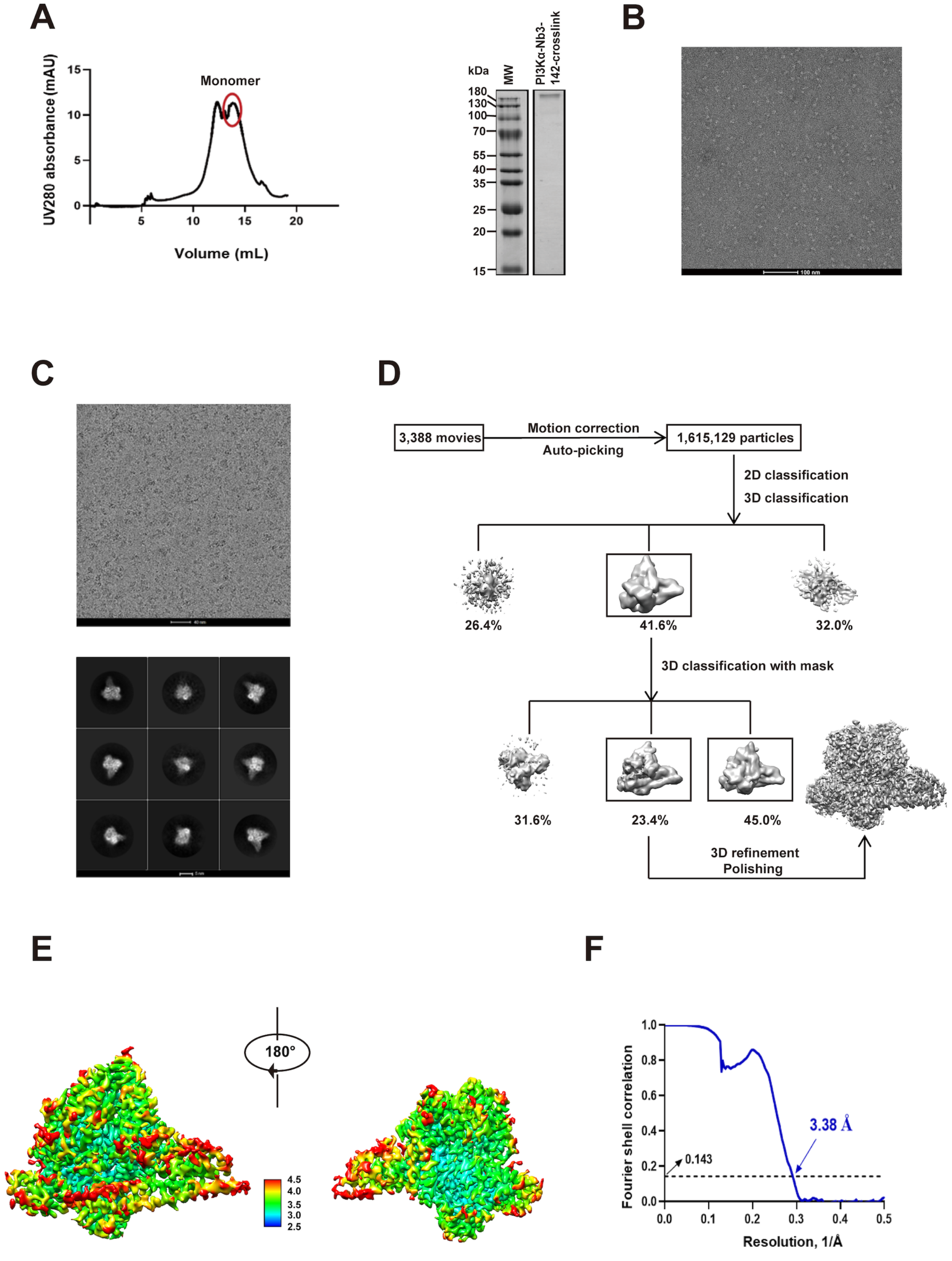
A
UV280 absorbance (mAU)
Monomer
Volume (mL)
kDa
MW
PI3Kα-Nb3-142-crosslink
180
130
100
70
55
40
35
25
20
15
B
C
D
3,388 movies
Motion correction
Auto-picking
1,615,129 particles
2D classification
3D classification
26.4%
41.6%
32.0%
3D classification with mask
31.6%
23.4%
45.0%
3D refinement
Polishing
E
180°
4.5
4.0
3.5
3.0
2.5
F
Fourier shell correlation
3.38 Å
0.143
Resolution, 1/Å

Fig. S8. Characterization and cryo-EM analysis of the DSG cross-linked Nb3-142 complex. (A) Analytical size exclusion chromatography, SDS-PAGE/Coomassie blue stain of the purified cross-linked Nb3-142 complex. (B) Representative negative staining image of the purified cross-linked Nb3-142 complex. (C) Representative cryo-EM micrographs of the cross-linked Nb3-142 complex. Representative 2D class averages showing distinct secondary structure features from different views. (D) Workflow of cryo-EM data processing for the cross-linked Nb3-142 complex. (E) Cryo-EM maps are colored by local resolution (Å). (F) 'Gold-standard' FSC curves of the cross-linked Nb3-142 complex, indicating the resolution is 3.38 Å at a FSC of 0.143.

Table S1. Inter-protein cross-links identified between PI3Kα and Nb3-126.

	**Nb3-126 Cross-link pair**	**Linker**	**Peptide sequence**	**Best E-value[a]**	**Best score[b]**
**1**	Nb3-126(1)-p110α(51)	BS^3, DSG	QVQLQESGGGLVQAGHSLR(1)-HELFKEAR(5)	7.22E-13	5.65E-01
**2**	Nb3-126(1)-p110α(100)	DSG	QVQLQESGGGLVQAGHSLR(1)-LFQPFLKVIEPVGNREEK(7)	8.32E-05	1.22E-02
**3**	Nb3-126(1)-p110α(111)	BS^3	QVQLQESGGGLVQAGHSLR(1)-VIEPVGNREEKILNR(11)	2.36E-06	8.73E-02
**4**	Nb3-126(1)-p110α(148)	BS^3, DSG	QVQLQESGGGLVQAGHSLR(1)-NILNVCKEAVDLR(7)	1.21E-07	1.93E-01
**5**	Nb3-126(1)-p110α(173)	BS^3	QVQLQESGGGLVQAGHSLR(1)-AMYVYPPNVESSPELPKHIYNK(11)	3.08E-05	1.18E-01
**6**	Nb3-126(1)-p110α(179)	BS^3	QVQLQESGGGLVQAGHSLR(1)-AMYVYPPNVESSPELPKHIYNK(17)	4.86E-05	1.11E-01
**7**	Nb3-126(1)-p110α(260)	BS^3	QVQLQESGGGLVQAGHSLR(1)-VCGCDEYFLEKYPLSQYK(7)	1.84E-04	3.27E-05
**8**	Nb3-126(1)-p110α(264)	DSG	QVQLQESGGGLVQAGHSLR(1)-VCGCDEYFLEKYPLSQYK(11)	6.89E-10	3.26E-04
**9**	Nb3-126(1)-p110α(323)	DSG	QVQLQESGGGLVQAGHSLR(1)-ISTATPYMNGETSTKSLWVINSALR(13)	6.86E-09	5.60E-04
**10**	Nb3-126(1)-p110α(324)	BS^3, DSG	QVQLQESGGGLVQAGHSLR(1)-ISTATPYMNGETSTKSLWVINSALR(14)	6.74E-10	4.50E-02
**11**	Nb3-126(1)-p110α(325)	BS^3, DSG	QVQLQESGGGLVQAGHSLR(1)-ISTATPYMNGETSTKSLWVINSALR(15)	1.96E-10	6.84E-03

			QVQLQESGGGLVQAGHSLR(1)-RISTATPYMNGETSTKSLWVINSALR(16)		
**12**	Nb3-126(1)-p110α(337)	BS^3	QVQLQESGGGLVQAGHSLR(1)-IKILCATYVNVNIR(2)	4.95E-06	3.89E-01
**13**	Nb3-126(1)-p110α(353)	BS^3, DSG	QVQLQESGGGLVQAGHSLR(1)-DIDKIYVR(4)	5.01E-04	1.08E-01
**14**	Nb3-126(1)-p110α(413)	BS^3	QVQLQESGGGLVQAGHSLR(1)-KGAKEEHCPLAWGNINLFDYTDTLVSGK(1)	4.45E-06	5.00E-03
**15**	Nb3-126(1)-p110α(416)	BS^3	QVQLQESGGGLVQAGHSLR(1)-KGAKEEHCPLAWGNINLFDYTDTLVSGK(4)	1.55E-11	8.05E-04
**16**	Nb3-126(1)-p110α(507)	BS^3, DSG	QVQLQESGGGLVQAGHSLR(1)-EAGFSYSHAGLSNR(5)	1.76E-08	3.50E-01
**17**	Nb3-126(1)-p110α(509)	BS^3	QVQLQESGGGLVQAGHSLR(1)-EAGFSYSHAGLSNR(7)	6.18E-05	1.57E-05
**18**	Nb3-126(1)-p110α(528)	BS^3, DSG	QVQLQESGGGLVQAGHSLR(1)-DNELRENDKEQLK(9) QVQLQESGGGLVQAGHSLR(1)-ENDKEQLK(4)	2.88E-06	3.69E-02
**19**	Nb3-126(1)-p110α(656)	BS^3	QVQLQESGGGLVQAGHSLR(1)-KALTNQR(1)	5.31E-06	5.60E-02
**20**	Nb3-126(1)-p110α(700)	DSG	QVQLQESGGGLVQAGHSLR(1)-ACGMYLKHLNR(7)	3.90E-07	6.36E-01
**21**	Nb3-126(1)-p110α(729)	BS^3	QVQLQESGGGLVQAGHSLR(1)-KDETQKVQMK(6)	4.48E-04	7.91E-04
**22**	Nb3-126(1)-p110α(733)	BS^3	QVQLQESGGGLVQAGHSLR(1)-VQMKFLVEQMR(4)	3.02E-09	1.52E-02
**23**	Nb3-126(1)-p110α(972)	BS^3	QVQLQESGGGLVQAGHSLR(1)-GAQECTK(6)	2.73E-05	4.39E-02
**24**	Nb3-126(1)-p110α(985)	BS^3, DSG	QVQLQESGGGLVQAGHSLR(1)-FQEMCYKAYLAIR(6)	6.96E-05	1.20E-01

**25**	Nb3-126(1)-p110α(986)	BS3	QVQLQESGGGLVQAGHSLR(1)-FQEMCYKAYLAIR(7)	1.80E-07	7.85E-03
**26**	Nb3-126(1)-p110α(1030)	BS3, DSG	QVQLQESGGGLVQAGHSLR(1)-TLALDKTEQEALEYFMK(6)	7.13E-14	3.44E-02
**27**	Nb3-126(1)-p110α(1041)	BS3	QVQLQESGGGLVQAGHSLR(1)-TLALDKTEQEALEYFMKQMNDAHHGGWTTK(17)	2.00E-06	1.53E-01
**28**	Nb3-126(1)-p110α(1063)	BS3	QVQLQESGGGLVQAGHSLR(1)-MDWIFHTIKQHALN(9)	9.79E-11	1.53E-03
**29**	Nb3-126(1)-p85α(14)	BS3, DSG	QVQLQESGGGLVQAGHSLR(1)-ALYDYKK(6)	8.99E-08	3.91E-01
**30**	Nb3-126(1)-p85α(15)	BS3	QVQLQESGGGLVQAGHSLR(1)-KEREEDIDLHLGDILTVNK(1)	2.46E-05	6.04E-02
**31**	Nb3-126(1)-p85α(80)	BS3, DSG	QVQLQESGGGLVQAGHSLR(1)-KISPPTPK(1) QVQLQESGGGLVQAGHSLR(1)-KKISPPTPK(2)	1.00E-11	3.65E-01
**32**	Nb3-126(1)-p85α(140)	BS3	QVQLQESGGGLVQAGHSLR(1)-LVEAIEKK(7)	1.56E-04	3.76E-01
**33**	Nb3-126(1)-p85α(141)	BS3, DSG	QVQLQESGGGLVQAGHSLR(1)-KGLECSTLYR(1)	2.71E-11	4.74E-02
**34**	Nb3-126(1)-p85α(254)	DSG	QVQLQESGGGLVQAGHSLR(1)-LSQTSSKNLLNAR(6)	4.69E-04	6.73E-01
**35**	Nb3-126(1)-p85α(255)	BS3	QVQLQESGGGLVQAGHSLR(1)-LSQTSSKNLLNAR(7)	1.62E-05	1.56E-03
**36**	Nb3-126(1)-p85α(361)	BS3	QVQLQESGGGLVQAGHSLR(1)-DASTKMHGDYTLTLR(4)	1.94E-14	6.96E-01
**37**	Nb3-126(1)-p85α(388)	BS3, DSG	QVQLQESGGGLVQAGHSLR(1)-DGKYGFSDPLTFSSVVELINHYR(3)	3.24E-15	3.60E-02
**38**	Nb3-126(1)-p85α(389)	BS3	QVQLQESGGGLVQAGHSLR(1)-YGFSDPLTFSSVVELINHYR(1)	2.21E-11	2.27E-03

**39**	Nb3-126(1)-p85α(392)	BS^3	QVQLQESGGGLVQAGHSLR(1)-YGFSDPLTFSSVVELINHYR(4)	4.63E-12	2.34E-03
**40**	Nb3-126(1)-p85α(398)	BS^3	QVQLQESGGGLVQAGHSLR(1)-YGFSDPLTFSSVVELINHYR(10)	1.03E-04	4.41E-03
**41**	Nb3-126(1)-p85α(491)	DSG	QVQLQESGGGLVQAGHSLR(1)-TAIEAFNETIKIFEEQCQTQER(11)	7.29E-22	2.18E-02
**42**	Nb3-126(1)-p85α(652)	BS^3	QVQLQESGGGLVQAGHSLR(1)-ESSKQGCYACSVVVDGEVK(4)	3.68E-08	5.03E-05
**43**	Nb3-126(1)-p85α(673)	BS^3	QVQLQESGGGLVQAGHSLR(1)-HCVINKTATGYGFAEPYNLYSSLK(6)	1.71E-09	5.05E-05
**44**	Nb3-126(1)-p85α(674)	BS^3, DSG	QVQLQESGGGLVQAGHSLR(1)-HCVINKTATGYGFAEPYNLYSSLK(7)	1.74E-19	1.40E-03
**45**	Nb3-126(1)-p85α(418)	BS^3, DSG	QVQLQESGGGLVQAGHSLR(1)-NESLAQYNPKLDVK(10)	7.28E-08	6.73E-01
**46**	Nb3-126(1)-p85α(422)	BS^3, DSG	QVQLQESGGGLVQAGHSLR(1)-LDVKLLYPVSK(4)	9.54E-12	5.07E-01
**47**	Nb3-126(1)-p85α(437)	BS^3, DSG	QVQLQESGGGLVQAGHSLR(1)-YQQDQVVKEDNIEAVGK(8) QVQLQESGGGLVQAGHSLR(1)-YQQDQVVKEDNIEAVGKK(8)	2.11E-11	6.77E-03
**48**	Nb3-126(1)-p85α(446)	BS^3	QVQLQESGGGLVQAGHSLR(1)-YQQDQVVKEDNIEAVGKK(17) QVQLQESGGGLVQAGHSLR(1)-EDNIEAVGKK(9)	1.73E-06	2.11E-01

**49**	Nb3-126(1)-p85α(447)	BS^3	QVQLQESGGGLVQAGHSLR(1)-KLHEYNTQFQEK(1)	1.45E-15	9.73E-03
**50**	Nb3-126(1)-p85α(458)	BS^3, DSG	QVQLQESGGGLVQAGHSLR(1)-LHEYNTQFQEKSR(11) QVQLQESGGGLVQAGHSLR(1)-KLHEYNTQFQEKSR(12)	6.08E-11	2.84E-01
**51**	Nb3-126(1)-p85α(504)	BS^3, DSG	QVQLQESGGGLVQAGHSLR(1)-YSKEYIEK(2)	1.54E-09	3.91E-01
**52**	Nb3-126(1)-p85α(529)	BS^3	QVQLQESGGGLVQAGHSLR(1)-IMHNYDKLK(7)	4.76E-08	2.00E-02
**53**	Nb3-126(1)-p85α(549)	BS^3, DSG	QVQLQESGGGLVQAGHSLR(1)-RLEEDLKK(7)	2.58E-08	4.66E-01
**54**	Nb3-126(1)-p85α(550)	BS^3, DSG	QVQLQESGGGLVQAGHSLR(1)-KQAAEYR(1)	9.35E-13	1.92E-01
**55**	Nb3-126(1)-p85α(566)	BS^3, DSG	QVQLQESGGGLVQAGHSLR(1)-MNSIKPDLIQLR(5)	5.48E-08	6.02E-01
**56**	Nb3-126(1)-p85α(584)	BS^3, DSG	QVQLQESGGGLVQAGHSLR(1)-DQYLMWLTQKGVR(8) QVQLQESGGGLVQAGHSLR(1)-TRDQYLMWLTQKGVR(10)	5.70E-07	1.86E-01
**57**	Nb3-126(1)-p85α(586)	BS^3, DSG	QVQLQESGGGLVQAGHSLR(1)-DQYLMWLTQKGVR(10) QVQLQESGGGLVQAGHSLR(1)-TRDQYLMWLTQKGVR(12)	1.35E-17	6.51E-01
**58**	Nb3-126(1)-p85α(632)	BS^3, DSG	QVQLQESGGGLVQAGHSLR(1)-NKAENLLR(2)	7.47E-11	6.34E-01
**59**	Nb3-126(25)-p110α(972)	DSG	LSCAASGR(6)-GAQECTKTR(6)	1.58E-08	3.28E-01
**60**	Nb3-126(25)-p85α(518)	DSG	LSCAASGR(6)-EGNEKEIQR(5)	3.84E-08	5.80E-01
**61**	Nb3-126(25)-p85α(632)	BS^3, DSG	LSCAASGR(6)-NKAENLLR(2)	8.78E-10	1.04E-01
**62**	Nb3-126(43)-p85α(14)	DSG	RAPGKER(5)-ALYDYKK(6) APGKER(4)-ALYDYKK(6)	2.73E-14	7.98E-01

**63**	Nb3-126(43)-p85α(80)	DSG	RAPGKER(5)-KISPPTPK(1)	1.23E-08	7.81E-01
**64**	Nb3-126(43)-p85α(549)	DSG	RAPGKER(5)-RLEEDLKK(7) RAPGKER(5)-LEEDLKK(6)	8.23E-10	6.66E-01
**65**	Nb3-126(43)-p85α(550)	DSG	RAPGKER(5)-KQAAEYR(1) APGKER(4)-KQAAEYR(1)	5.57E-05	7.88E-01
**66**	Nb3-126(43)-p85α(632)	DSG	RAPGKER(5)-NKAENLLR(2) APGKER(4)-NKAENLLR(2)	3.01E-08	7.43E-01
**67**	Nb3-126(52)-p110α(51)	BS^3	EFVAGISKGENYR(7)-HELFKEAR(5)	4.17E-13	4.43E-01
**68**	Nb3-126(52)-p110α(353)	BS^3, DSG	EFVAGISKGENYR(7)-DIDKIYVR(4)	3.57E-05	4.25E-01
**69**	Nb3-126(52)-p110α(416)	BS^3	EFVAGISKGENYR(7)-KGAKEEHCPLAWGNINLFDYTDTLVSGK(4)	1.47E-07	1.37E-02
**70**	Nb3-126(52)-p110α(507)	BS^3	EFVAGISKGENYR(7)-EAGFSYSHAGLSNR(5)	6.76E-07	2.80E-01
**71**	Nb3-126(52)-p110α(528)	DSG	EFVAGISKGENYR(7)-DNELRENDKEQLK(9)	7.33E-04	5.94E-01
**72**	Nb3-126(52)-p110α(985)	BS^3	EFVAGISKGENYR(7)-FQEMCYKAYLAIR(6)	4.44E-04	9.24E-02
**73**	Nb3-126(52)-p110α(986)	BS^3, DSG	EFVAGISKGENYR(7)-FQEMCYKAYLAIR(7)	1.38E-09	6.57E-01
**74**	Nb3-126(52)-p110α(1061)	BS^3	EFVAGISKGENYR(7)-MDWIFHTIKQHALN(7)	5.73E-05	1.15E-02
**75**	Nb3-126(52)-p110α(1063)	BS^3	EFVAGISKGENYR(7)-MDWIFHTIKQHALN(9)	1.48E-05	3.32E-01
**76**	Nb3-126(52)-p85α(80)	BS^3	EFVAGISKGENYR(7)-KISPPTPK(1) EFVAGISKGENYR(7)-KKISPPTPK(2)	1.96E-05	2.49E-01
**77**	Nb3-126(52)-p85α(287)	DSG	EFVAGISKGENYR(7)-FSAASSDNTENLIKVIEILISTEWNER(14)	6.62E-04	5.08E-01
**78**	Nb3-126(52)-p85α(504)	BS^3, DSG	EFVAGISKGENYR(7)-YSKEYIEK(2)	1.25E-07	2.06E-01
**79**	Nb3-126(52)-p85α(505)	BS^3	EFVAGISKGENYR(7)-YSKEYIEK(3)	1.33E-04	1.92E-03
**80**	Nb3-126(52)-p85α(518)	BS^3	EFVAGISKGENYR(7)-EGNEKEIQR(5)	3.21E-04	3.14E-01

**81**	Nb3-126(52)-p85α(529)	BS^3, DSG	EFVAGISKGENYR(7)-IMHNYDKLK(7)	6.47E-07	5.56E-01
**82**	Nb3-126(52)-p85α(550)	DSG	EFVAGISKGENYR(7)-KQAAEYR(1)	8.58E-18	4.84E-01
**83**	Nb3-126(52)-p85α(632)	BS^3, DSG	EFVAGISKGENYR(7)-NKAENLLR(2)	9.18E-06	3.33E-01
**84**	Nb3-126(53)-p110α(51)	BS^3	EFVAGISKGENYR(8)-HELFKEAR(5)	8.80E-13	1.54E-01
**85**	Nb3-126(53)-p110α(353)	BS^3, DSG	EFVAGISKGENYR(8)-DIDKIYVR(4)	2.20E-07	8.15E-01
**86**	Nb3-126(53)-p110α(416)	BS^3	EFVAGISKGENYR(8)-KGAKEEHCPLAWGNINLFDYTDTLVSGK(4)	7.50E-05	4.11E-01
**87**	Nb3-126(53)-p110α(972)	BS^3	EFVAGISKGENYR(8)-GAQECTKTR(6)	9.70E-04	4.48E-01
**88**	Nb3-126(53)-p110α(985)	BS^3, DSG	EFVAGISKGENYR(8)-FQEMCYKAYLAIR(6)	5.60E-11	1.65E-01
**89**	Nb3-126(53)-p110α(986)	BS^3, DSG	EFVAGISKGENYR(8)-FQEMCYKAYLAIR(7)	1.01E-12	2.62E-04
**90**	Nb3-126(53)-p85α(14)	BS^3, DSG	EFVAGISKGENYR(8)-ALYDYKK(6)	2.26E-06	2.02E-01
**91**	Nb3-126(53)-p85α(80)	BS^3	EFVAGISKGENYR(8)-KISPPTPK(1)	7.07E-05	2.17E-01
**92**	Nb3-126(53)-p85α(381)	BS^3	EFVAGISKGENYR(8)-LIKIFHR(3)	1.35E-06	5.50E-01
**93**	Nb3-126(53)-p85α(447)	BS^3	EFVAGISKGENYR(8)-KLHEYNTQFQEK(1)	6.65E-04	4.92E-01
**94**	Nb3-126(53)-p85α(503)	DSG	EFVAGISKGENYR(8)-YSKEYIEK(1)	5.09E-04	4.60E-02
**95**	Nb3-126(53)-p85α(504)	DSG	EFVAGISKGENYR(8)-YSKEYIEK(2)	9.01E-08	6.62E-03
**96**	Nb3-126(53)-p85α(505)	BS^3	EFVAGISKGENYR(8)-YSKEYIEK(3)	1.33E-06	7.98E-03
**97**	Nb3-126(53)-p85α(518)	BS^3, DSG	EFVAGISKGENYR(8)-EGNEKEIQR(5)	1.04E-04	6.21E-01
**98**	Nb3-126(53)-p85α(529)	BS^3, DSG	EFVAGISKGENYR(8)-IMHNYDKLK(7)	9.61E-07	1.40E-01
**99**	Nb3-126(53)-p85α(550)	DSG	EFVAGISKGENYR(8)-KQAAEYR(1)	3.22E-10	7.37E-01
**100**	Nb3-126(53)-p85α(632)	BS^3, DSG	EFVAGISKGENYR(8)-NKAENLLR(2)	1.79E-10	8.21E-02
**101**	Nb3-126(63)-p110α(51)	DSG	SYADSVKGR(5)-HELFKEAR(5)	5.75E-08	4.95E-01
**102**	Nb3-126(63)-p85α(437)	DSG	SYADSVKGR(5)-YQQDQVVKEDNIEAVGK(8)	1.84E-05	4.86E-01

**103**	Nb3-126(65)-p110α(353)	BS3, DSG	SYADSVKGR(7)-DIDKIYVR(4)	8.95E-12	8.04E-01
**104**	Nb3-126(65)-p110α(973)	DSG	SYADSVKGR(7)-GAQECTKTR(7)	1.60E-15	2.98E-01
**105**	Nb3-126(65)-p85α(14)	DSG	SYADSVKGR(7)-ALYDYKK(6)	2.10E-11	6.97E-01
**106**	Nb3-126(65)-p85α(254)	DSG	SYADSVKGR(7)-LSQTSSKNLLNAR(6)	1.94E-04	2.27E-01
**107**	Nb3-126(65)-p85α(446)	DSG	SYADSVKGR(7)-EDNIEAVGKK(9)	1.03E-08	2.96E-01
**108**	Nb3-126(65)-p85α(504)	BS3, DSG	SYADSVKGR(7)-YSKEYIEK(2)	3.73E-05	3.79E-01
**109**	Nb3-126(65)-p85α(518)	DSG	SYADSVKGR(7)-EGNEKEIQR(5)	1.75E-13	2.98E-01
**110**	Nb3-126(65)-p85α(550)	DSG	SYADSVKGR(7)-KQAAEYR(1)	3.95E-04	4.72E-01
**111**	Nb3-126(65)-p85α(632)	BS3, DSG	SYADSVKGR(7)-NKAENLLR(2)	3.77E-14	6.22E-01
**112**	Nb3-126(65)-p85α(650)	DSG	SYADSVKGR(7)-ESSKQGCYACSVVVDGEVK(2)	2.34E-04	1.59E-02
**113**	Nb3-126(76)-p110α(353)	DSG	DNAKSTVYLQMNSLKPDDAAVYYCAAK(4)-DIDKIYVR(4)	5.95E-04	8.12E-01
**114**	Nb3-126(76)-p85α(388)	DSG	DNAKSTVYLQMNSLKPDDAAVYYCAAK(4)-DGKYGFSDPLTFSSVVELINHYR(3)	2.19E-08	3.17E-05
**115**	Nb3-126(85)-p85α(632)	DSG	STVYLQMNSLKPDDAAVYYCAAK(9)-NKAENLLR(2)	1.76E-06	5.23E-01

[a]E-value means normalized refined_scores for filtered peptide spectrum matches (PSMs) by FDR, the smaller the more confident. Best E-value is the smallest E-value of all spectra of the cross-linked peptide. [b]Score, which is short for SVM score in pLink 2, is the prime measure for FDR estimation. Best score is the smallest value of all spectra of the cross-linked peptide.

Table S2. Inter-protein cross-links identified between PI3Kα and Nb3-142.

	Nb3-142 Cross-link pair	Linker	Peptide sequence	Best E-value[a]	Best score[b]
**1**	Nb3-142(43)-p85α(632)	DSG	QTPGKER(5)-NKAENLLR(2)	6.58E-05	6.97E-01
**2**	Nb3-142(60)-p85α(80)	DSG	YADSVKGR(1)-KISPPTPK(1)	8.59E-04	4.86E-01
**3**	Nb3-142(63)-p110α(51)	BS^3	YADSVKGR(4)-HELFKEAR(5)	1.65E-06	8.14E-01
**4**	Nb3-142(63)-p85α(418)	DSG	YADSVKGR(4)-NESLAQYNPKLDVK(10)	1.94E-06	5.34E-01
**5**	Nb3-142(65)-p110α(51)	DSG	YADSVKGR(6)-HELFKEAR(5)	6.13E-09	4.58E-01
**6**	Nb3-142(65)-p110α(353)	BS^3, DSG	YADSVKGR(6)-DIDKIYVR(4)	5.75E-13	4.27E-01
**7**	Nb3-142(65)-p110α(973)	DSG	YADSVKGR(6)-GAQECTKTR(7)	7.79E-15	8.02E-02
**8**	Nb3-142(65)-p85α(14)	BS^3, DSG	YADSVKGR(6)-ALYDYKK(6)	5.33E-11	5.17E-01
**9**	Nb3-142(65)-p85α(80)	BS^3, DSG	YADSVKGR(6)-KISPPTPK(1)	2.94E-07	8.16E-01
**10**	Nb3-142(65)-p85α(418)	DSG	YADSVKGR(6)-NESLAQYNPKLDVK(10)	1.98E-07	4.21E-01
**11**	Nb3-142(65)-p85α(422)	DSG	YADSVKGR(6)-LDVKLLYPVSK(4)	8.29E-04	2.10E-01
**12**	Nb3-142(65)-p85α(549)	DSG	YADSVKGR(6)-LEEDLKK(6)	1.62E-07	6.66E-01
**13**	Nb3-142(65)-p85α(550)	DSG	YADSVKGR(6)-KQAAEYR(1)	5.37E-08	3.54E-01
**14**	Nb3-142(65)-p85α(632)	BS^3, DSG	YADSVKGR(6)-NKAENLLR(2)	1.97E-15	6.94E-01
**15**	Nb3-142(65)-p85α(650)	DSG	YADSVKGR(6)-ESSKQGCYACSVVVDGEVK(2)	3.96E-04	9.66E-02
**16**	Nb3-142(65)-p85α(651)	DSG	YADSVKGR(6)-ESSKQGCYACSVVVDGEVK(3)	6.11E-04	1.86E-01

[a]E-value means normalized refined_scores for filtered peptide spectrum matches (PSMs) by FDR, the smaller the more confident. Best E-value is the smallest E-value of all spectra of the cross-linked peptide. [b]Score, which is short for SVM score in pLink 2, is the prime measure for FDR estimation. Best score is the smallest value of all spectra of the cross-linked peptide.

Table S3. Inter-protein cross-links identified between PI3Kα and Nb3-159.

	Nb3-159 Cross-link pair	Linker	Peptide sequence	Best E-value[a]	Best score[b]
**1**	Nb3-159(1)-p110α(51)	BS^3, DSG	QVQLQESGGGLVQTGGSLR(1)-HELFKEAR(5)	1.75E-12	5.29E-01
**2**	Nb3-159(1)-p110α(353)	BS^3,	QVQLQESGGGLVQTGGSLR(1)-DIDKIYVR(4)	2.40E-04	5.56E-02
**3**	Nb3-159(1)-p110α(507)	BS^3, DSG	QVQLQESGGGLVQTGGSLR(1)-EAGFSYSHAGLSNR(5)	1.17E-07	1.78E-02
**4**	Nb3-159(1)-p110α(528)	BS^3, DSG	QVQLQESGGGLVQTGGSLR(1)-DNELRENDKEQLK(9)	1.63E-05	2.78E-02
**5**	Nb3-159(1)-p110α(532)	DSG	QVQLQESGGGLVQTGGSLR(1)-ENDKEQLKAISTR(8)	1.33E-05	5.67E-01
**6**	Nb3-159(1)-p110α(973)	BS^3	QVQLQESGGGLVQTGGSLR(1)-GAQECTKTR(7)	5.24E-04	3.26E-04
**7**	Nb3-159(1)-p110α(1063)	BS^3, DSG	QVQLQESGGGLVQTGGSLR(1)-MDWIFHTIKQHALN(9)	9.30E-11	8.14E-01
**8**	Nb3-159(1)-p85α(14)	BS^3	QVQLQESGGGLVQTGGSLR(1)-ALYDYKK(6)	2.41E-05	2.25E-02
**9**	Nb3-159(1)-p85α(80)	BS^3, DSG	QVQLQESGGGLVQTGGSLR(1)-KISPPTPK(1) QVQLQESGGGLVQTGGSLR(1)-KKISPPTPK(2)	9.89E-11	4.84E-01
**10**	Nb3-159(1)-p85α(253)	BS^3	QVQLQESGGGLVQTGGSLR(1)-LSQTSSKNLLNAR(5)	9.35E-04	1.09E-03
**11**	Nb3-159(1)-p85α(254)	BS^3	QVQLQESGGGLVQTGGSLR(1)-LSQTSSKNLLNAR(6)	5.78E-05	1.85E-02
**12**	Nb3-159(1)-p85α(255)	BS^3	QVQLQESGGGLVQTGGSLR(1)-LSQTSSKNLLNAR(7)	2.32E-04	1.27E-01
**13**	Nb3-159(1)-p85α(361)	DSG	QVQLQESGGGLVQTGGSLR(1)-DASTKMHGDYTLTLR(4)	1.06E-04	2.65E-02
**14**	Nb3-159(1)-p85α(381)	BS^3, DSG	QVQLQESGGGLVQTGGSLR(1)-LIKIFHR(3)	4.94E-09	6.65E-01
**15**	Nb3-159(1)-p85α(389)	BS^3	QVQLQESGGGLVQTGGSLR(1)-YGFSDPLTFSSVVELINHYR(1)	9.37E-12	5.51E-13

**16**	Nb3-159(1)-p85α(392)	BS^3	QVQLQESGGGLVQTGGSLR(1)-YGFSDPLTFSSVVELINHYR(4)	5.63E-04	1.14E-04
**17**	Nb3-159(1)-p85α(398)	BS^3	QVQLQESGGGLVQTGGSLR(1)-YGFSDPLTFSSVVELINHYR(10)	7.53E-05	7.82E-02
**18**	Nb3-159(1)-p85α(399)	BS^3	QVQLQESGGGLVQTGGSLR(1)-YGFSDPLTFSSVVELINHYR(11)	5.87E-09	7.18E-02
**19**	Nb3-159(1)-p85α(418)	BS^3, DSG	QVQLQESGGGLVQTGGSLR(1)-NESLAQYNPKLDVK(10)	1.96E-09	1.28E-04
**20**	Nb3-159(1)-p85α(422)	BS^3, DSG	QVQLQESGGGLVQTGGSLR(1)-LDVKLLYPVSK(4)	6.27E-09	9.73E-02
**21**	Nb3-159(1)-p85α(437)	BS^3, DSG	QVQLQESGGGLVQTGGSLR(1)-YQQDQVVKEDNIEAVGK(8)<br>QVQLQESGGGLVQTGGSLR(1)-YQQDQVVKEDNIEAVGKK(8)	2.30E-13	5.17E-04
**22**	Nb3-159(1)-p85α(446)	BS^3, DSG	QVQLQESGGGLVQTGGSLR(1)-YQQDQVVKEDNIEAVGKK(17)<br>QVQLQESGGGLVQTGGSLR(1)-EDNIEAVGKK(9)	1.93E-08	1.57E-02
**23**	Nb3-159(1)-p85α(447)	DSG	QVQLQESGGGLVQTGGSLR(1)-KLHEYNTQFQEK(1)	9.61E-06	2.85E-02
**24**	Nb3-159(1)-p85α(458)	DSG	QVQLQESGGGLVQTGGSLR(1)-LHEYNTQFQEKSR(11)<br>QVQLQESGGGLVQTGGSLR(1)-KLHEYNTQFQEKSR(12)	2.20E-11	4.31E-01
**25**	Nb3-159(1)-p85α(504)	DSG	QVQLQESGGGLVQTGGSLR(1)-YSKEYIEK(2)	4.40E-07	6.69E-01
**26**	Nb3-159(1)-p85α(529)	BS^3, DSG	QVQLQESGGGLVQTGGSLR(1)-IMHNYDKLK(7)	1.08E-05	7.16E-01
**27**	Nb3-159(1)-p85α(549)	DSG	QVQLQESGGGLVQTGGSLR(1)-LEEDLKK(6)	1.34E-08	6.41E-01

			QVQLQESGGGLVQTGGSLR(1)-RLEEDLKK(7)		
**28**	Nb3-159(1)-p85α(550)	BS^3	QVQLQESGGGLVQTGGSLR(1)-KQAAEYR(1)	3.63E-12	2.43E-03
**29**	Nb3-159(1)-p85α(566)	DSG	QVQLQESGGGLVQTGGSLR(1)-MNSIKPDLIQLR(5)	1.30E-08	1.45E-02
**30**	Nb3-159(1)-p85α(586)	DSG	QVQLQESGGGLVQTGGSLR(1)-DQYLMWLTQKGVR(10) QVQLQESGGGLVQTGGSLR(1)-TRDQYLMWLTQKGVR(12)	2.34E-23	2.71E-01
**31**	Nb3-159(1)-p85α(632)	BS^3, DSG	QVQLQESGGGLVQTGGSLR(1)-NKAENLLR(2)	2.11E-09	2.52E-01
**32**	Nb3-159(1)-p85α(674)	BS^3, DSG	QVQLQESGGGLVQTGGSLR(1)-HCVINKTATGYGFAEPYNLYSSLK(7)	3.45E-13	5.09E-03
**33**	Nb3-159(43)-p110α(353)	BS^3, DSG	QAPGKER(5)-DIDKIYVR(4)	3.86E-06	8.14E-01
**34**	Nb3-159(43)-p85α(550)	DSG	QAPGKER(5)-KQAAEYR(1)	6.99E-08	2.45E-01
**35**	Nb3-159(43)-p85α(632)	BS^3, DSG	QAPGKER(5)-NKAENLLR(2)	7.75E-06	8.07E-01
**36**	Nb3-159(63)-P110α(51)	BS^3, DSG	GSNTSYADSVKGR(9)-HELFKEAR(5)	4.58E-06	4.21E-01
**37**	Nb3-159(63)-p110α(353)	DSG	GSNTSYADSVKGR(9)-DIDKIYVR(4)	3.11E-07	3.52E-01
**38**	Nb3-159(63)-p85α(80)	BS^3, DSG	GSNTSYADSVKGR(9)-KISPPTPK(1) GSNTSYADSVKGR(9)-KKISPPTPK(2)	3.42E-08	8.15E-01
**39**	Nb3-159(63)-p85α(422)	DSG	GSNTSYADSVKGR(9)-LDVKLLYPVSK(4)	5.15E-10	4.07E-01
**40**	Nb3-159(63)-p85α(437)	DSG	GSNTSYADSVKGR(9)-YQQDQVVKEDNIEAVGKK(8)	7.40E-05	4.92E-01
**41**	Nb3-159(63)-p85α(458)	DSG	GSNTSYADSVKGR(9)-LHEYNTQFQEKSR(11)	3.18E-05	6.74E-01
**42**	Nb3-159(63)-p85α(549)	DSG	GSNTSYADSVKGR(9)-LEEDLKK(6)	7.39E-14	2.71E-01
**43**	Nb3-159(63)-p85α(586)	DSG	GSNTSYADSVKGR(9)-DQYLMWLTQKGVR(10)	5.61E-11	1.08E-01

**44**	Nb3-159(63)-p85α(673)	DSG	GSNTSYADSVKGR(9)-HCVINKTATGYGFAEPYNLYSSLK(6)	7.16E-07	1.68E-01
**45**	Nb3-159(65)-p85α(80)	BS3, DSG	GSNTSYADSVKGR(11)-KISPPTPK(1) GSNTSYADSVKGR(11)-KKISPPTPK(2)	2.27E-12	8.02E-01
**46**	Nb3-159(65)-p110α(353)	BS3, DSG	GSNTSYADSVKGR(11)-DIDKIYVR(4)	3.56E-14	5.92E-01
**47**	Nb3-159(65)-p85α(422)	DSG	GSNTSYADSVKGR(11)-LDVKLLYPVSK(4)	4.58E-08	1.71E-03
**48**	Nb3-159(65)-p85α(446)	DSG	GSNTSYADSVKGR(11)-EDNIEAVGKK(9)	6.72E-06	6.42E-01
**49**	Nb3-159(65)-p85α(549)	DSG	GSNTSYADSVKGR(11)-LEEDLKK(6) GSNTSYADSVKGR(11)-RLEEDLKK(7)	4.12E-13	6.88E-01
**50**	Nb3-159(65)-p85α(550)	BS3, DSG	GSNTSYADSVKGR(11)-KQAAEYR(1)	5.41E-14	1.98E-01
**51**	Nb3-159(65)-p85α(586)	DSG	GSNTSYADSVKGR(11)-DQYLMWLTQKGVR(10)	4.97E-07	5.79E-01
**52**	Nb3-159(65)-p85α(632)	BS3, DSG	GSNTSYADSVKGR(11)-NKAENLLR(2)	1.24E-17	7.87E-01
**53**	Nb3-159(65)-p85α(650)	DSG	GSNTSYADSVKGR(11)-ESSKQGCYACSVVVDGEVK(2)	5.38E-09	7.37E-01
**54**	Nb3-159(76)-p85α(632)	BS3, DSG	DNTKNTVALQMNSLKPEDTAVYYCAADFGR(4)-NKAENLLR(2)	3.22E-05	7.85E-01
**55**	Nb3-159(85)-p85α(632)	DSG	NTVALQMNSLKPEDTAVYYCAADFGR(9)-NKAENLLR(2)	8.40E-07	5.48E-01
**56**	Nb3-159(87)-p85α(632)	BS3	NTVALQMNSLKPEDTAVYYCAADFGR(11)-NKAENLLR(2)	1.48E-06	1.19E-04

[a]E-value means normalized refined_scores for filtered peptide spectrum matches (PSMs) by FDR, the smaller the more confident. Best E-value is the smallest E-value of all spectra of the cross-linked peptide. [b]Score, which is short for SVM score in pLink 2, is the prime measure for FDR estimation. Best score is the smallest value of all spectra of the cross-linked peptide.

Table S4. Cryo-EM data acquisition and image processing.

**Data collection**				
Microscope	Titan Krios			
Voltage (kV)	300			
Corrected magnification	46,685			
Pixel size (Å)	1.071			
Detector	Gatan K3 Summit			
Defocus range (µm)	-1.5 to -2.5			
Defocus mean (µm)	-2.0			
Total electron exposure (e-$Å^{-2}s^{-1}$)	70			
Exposure rate (e-$Å^{-2}frame^{-1}$)	25			
**Data processing**	**PI3Kα–Nb3–126**	**PI3Kα–Nb3–142**	**PI3Kα–Nb3–159**	**PI3Kα–Nb3–142 (cross-linked)**
Useable image movies	4,397	5,211	3,012	3,388
Particles	4,952,325	7,049,901	2,882,334	1,615,129
Final particles	506,412	739,753	240,599	217,230
Map sharpening B factor ($Å^2$)	-81.3	-163.2	-144.3	-130.3
Resolution (Å)	2.41	3.08	3.13	3.38
Unmasked (0.143 FSC)	2.4	3.2	2.8	3.6
Masked (0.143 FSC)	2.4	3.1	2.8	3.4
Local resolution range (Å)	2.1-9.5	2.0-10.2	2.0-10.7	2.0-11.4
**Model**	**PI3Kα–Nb3–126**	**PI3Kα–Nb3–142**	**PI3Kα–Nb3–159**	**PI3Kα–Nb3–142 (cross-linked)**
EMDB accession code	EMD-27327	EMD-27334	EMD-27330	EMD-27336
PDB accession code	8DCP	8DD4	8DCX	8DD8
**Model composition**				
Chain	3	3	3	3
Non-hydrogen atom	10,620	10,425	9,646	10,727
Protein residue	1,284	1,255	1,169	1,300
**Refinement**				
Software	phenix.real_space_refine			
Resolution (Å)	2.3	3.1	3.1	3.5
CC (mask)	0.82	0.74	0.66	0.76

CC (peak)	0.70	0.70	0.51	0.74
CC (volume)	0.79	0.74	0.62	0.75
**Average B factor (Å²)**				
Protein	46.44	52.81	51.24	63.46
Ligand	19.91	33.94	---	---
**R.M.S. deviation**				
Bond length (Å) (# > 4σ)	0.003 (0)	0.003 (0)	0.002 (0)	0.003 (0)
Bond angle (°) (# > 4σ)	0.641 (0)	0.679 (1)	0.559 (1)	0.619 (1)
**Validation**				
Molprobity score	2.26	1.68	1.75	1.94
Clashscore, all atoms	9.43	10.00	9.76	11.24
Rotamer outlier (%)	3.64	0.00	0.00	0.00
Cβ outlier (%)	0.00	0.00	0.00	0.00
CaBLAM outlier (%)	2.70	1.63	2.24	3.33
**Ramachandran plot (%)**				
Outlier	0.00	0.16	0.09	0.31
Allowed	4.80	2.74	3.50	5.21
Favored	95.2	97.10	96.41	94.84

Dataset S1. CXMS derived restraints and docking analysis for nanobody PI3Kα complexes.
